# Tie2-mediated vascular remodeling by ferritin-based protein C nanoparticles confers antitumor and anti-metastatic activities

**DOI:** 10.1186/s13045-020-00952-9

**Published:** 2020-09-14

**Authors:** Young Sun Choi, Hyeonha Jang, Biki Gupta, Ji-Hak Jeong, Yun Ge, Chul Soon Yong, Jong Oh Kim, Jong-Sup Bae, Im-Sook Song, In-San Kim, You Mie Lee

**Affiliations:** 1 BK21 Plus KNU Multi-Omics Creative Drug Research Team, Daegu, Republic of Korea; 2 Department of Molecular Pathophysiology, Vessel-Organ Interaction Research Center, VOICE (MRC), College of Pharmacy, Kyungpook National University, Daegu, 41566 Republic of Korea; 3 Nano-Bio Application Team, National Nanofab Center (NNFC), 291 Daehak-ro, Yuseong-gu, Daejeon, 34141 Republic of Korea; 4 Research Institute of Pharmaceutical Sciences, Kyungpook National Univ., Daegu, Republic of Korea; 5 Present address Department of Pathology, University of Pittsburgh, 200 Lothrop Street, Pittsburgh, PA 15261 USA; 6 College of Pharmacy, Yeungnam University, Gyeongsan, 38541 Republic of Korea; 7 Biomedical Research Institute, Korea Institute of Science and Technology, Seoul, 02792 Republic of Korea

**Keywords:** Antitumor immune response, EPCR, Ferritin-based protein C nanoparticles, Tie2, Vascular normalization

## Abstract

**Background:**

Conventional therapeutic approaches for tumor angiogenesis, which are primarily focused on the inhibition of active angiogenesis to starve cancerous cells, target the vascular endothelial growth factor signaling pathway. This aggravates hypoxia within the tumor core and ultimately leads to increased tumor proliferation and metastasis. To overcome this limitation, we developed nanoparticles with antiseptic activity that target tumor vascular abnormalities.

**Methods:**

Ferritin-based protein C nanoparticles (PCNs), known as TFG and TFMG, were generated and tested in Lewis lung carcinoma (LLC) allograft and MMTV-PyMT spontaneous breast cancer models. Immunohistochemical analysis was performed on tumor samples to evaluate the tumor vasculature. Western blot and permeability assays were used to explore the role and mechanism of the antitumor effects of PCNs in vivo. For knocking down proteins of interest, endothelial cells were transfected with siRNAs. Statistical analysis was performed using one-way ANOVA followed by post hoc Dunnett’s multiple comparison test.

**Results:**

PCNs significantly inhibited hypoxia and increased pericyte coverage, leading to the inhibition of tumor growth and metastasis, while increasing survival in LLC allograft and MMTV-PyMT spontaneous breast cancer models. The coadministration of cisplatin with PCNs induced a synergistic suppression of tumor growth by improving drug delivery as evidenced by increased blood prefusion and decreased vascular permeability. Moreover, PCNs altered the immune cell profiles within the tumor by increasing cytotoxic T cells and M1-like macrophages with antitumor activity. PCNs induced PAR-1/PAR-3 heterodimerization through EPCR occupation and PAR-1 activation, which resulted in Gα13-RhoA-mediated-Tie2 activation and stabilized vascular tight junctions via the Akt-FoxO3a signaling pathway.

**Conclusions:**

Cancer treatment targeting the tumor vasculature by inducing antitumor immune responses and enhancing the delivery of a chemotherapeutic agent with PCNs resulted in tumor regression and may provide an effective therapeutic strategy.

## Background

Tumor-associated blood vessels exhibit prominent structural and functional abnormalities [1, 2]. The impaired and abnormal vasculature creates a tumor-promoting hypoxic microenvironment that promotes tumor progression, metastasis, and poor drug delivery [3, 4]. Conventional approaches have focused primarily on the inhibition of angiogenesis to starve cancerous cells [5, 6]. These agents primarily target vascular endothelial growth factor (VEGF) and its receptor, VEGFR2 [5, 6], resulting in an initial reduction of the existing tumor vessels. However, these treatments eventually aggravate hypoxia within the tumor core, triggering other proangiogenic signaling pathways that lead to increased tumor proliferation and metastasis [7, 8]. To avoid the side effects of conventional therapeutics, remodeling the tumor vasculature is a new approach that has been suggested to be an effective treatment strategy. Normalization of the tumor vasculature delivers increased anticancer benefits. These include efficient drug delivery, infiltration of immune cells by reducing the heterogeneity of intratumoral blood flow, and increased vascular permeability caused by tight associations between endothelial cells and perivascular cells [9, 10].

Recently, we introduced anti-inflammatory nanoparticles into the vasculature which utilize activated protein C (APC) [11]. The cytoprotective and anti-inflammatory activity of APC is mediated through binding to its receptor, endothelial cell protein C receptor (EPCR). This occurs through the *γ*-carboxyglutamic acid (Gla) domain and results in simultaneous activation of protease-activated receptor-1 (PAR-1) signaling via the thrombin receptor agonist peptide (TRAP) domain [12, 13]. APC also exhibits anti-coagulation activity. While the antithrombotic activity of APC is based on its ability to inactivate clotting factors Va and VIIIa, leading to adverse bleeding, the cytoprotective effect is based on its anti-inflammatory, antiapoptotic, and endothelial barrier stabilization activities [12, 14].

Since the activation of PAR-1 signaling by TRAP mimics thrombin activity [15] and results in a cytoprotective response when EPCR is occupied with APCs [15, 16], both the PC-Gla and TRAP peptides were attached to the surface of ferritin nanoparticles [11]. Ferritin nanoparticles are useful biocompatible, biodegradable, and nontoxic platforms compared with synthetic polymers [17], and ferritin-binding sites and ferritin internalization have been identified in some tumor cells [18]. Therefore, we used small ferritin as a basic template to create nanoparticles [11]. The PCNs consist of both EPCR-targeting peptides (PC-Gla) and PAR-1-activating peptides (TRAP) on their surface. A TRAP-small ferritin (sFn)-PC-Gla (TFG) or a matrix metalloproteinase (MMP)-2 cleavage site is inserted between sFn and the PC-Gla domain (TFMG). As MMP-2 is overexpressed in inflammatory sites as well as in the tumor microenvironment [19], PC-Gla is released upon reaching MMP-2 activating sites. Free PC-Gla will not likely interfere with the remaining TRAP-ferritin during the simultaneous double binding to each receptor by the TFMG nanoparticle [11]. Therefore, these ferritin-based protein cage protein C nanoparticles (PCNs) have simultaneous double occupancy of EPCR and PAR-1, but lack the ability to degrade procoagulant co-factors. Thus, they exhibit high therapeutic efficacy without causing bleeding complications [11]. Since cancer therapeutic research using nanoparticles to target the tumor vasculature has not been largely explored, we investigated the antitumor activity of PCNs through a new approach which results in the inhibition of tumor progression and metastasis.

In the present study, we used Lewis lung carcinoma (LLC) allograft and *MMTV*-*PyMT* spontaneous breast cancer models to demonstrate that PCNs exhibit antitumor and anti-metastatic activity. We found that the PCNs remodeled tumor vessels by increased pericyte coverage and led to improved tumor permeability, increased cisplatin cytotoxicity, and induced normalization of the tumor vasculature. In addition, we found that the PCNs significantly decreased the hypoxic area while increasing blood perfusion of tumor vessels by activating EPCR/PAR-1 and Tie2 simultaneously. This ameliorated the immune response as evidenced by increased antitumor T cell infiltration and decreased M2-like tumor-associated macrophages (TAM). Thus, our studies provide a foundation for the development of therapeutic strategies using nanoparticles that target tumor vascular normalization with antiseptic activity.

## Methods

### Materials

Bevacizumab was purchased from (InvivoGen, California, USA) and cisplatin (*cis*-diammineplatinum (II) dichloride, Cis), isopropyl-β-d-1-thiogalactopyranoside (IPTG), imidazole, and dithiothreitol (DTT) were purchased from Sigma-Aldrich (St Louis, MO, USA). Tris-HCl and Triton X-100 were purchased from Amresco (Solon, OH, USA), sodium chloride and urea from Junsei Chemical (Tokyo, Japan), and phenylmethane sulfonyl fluoride (PMSF) and protease inhibitor cocktail tablets from Roche (Basel, Switzerland). Mouse monoclonal antibodies against α-smooth muscle actin (SMA), pimonidazole, and cytokeratin II were purchased from Abcam (Cambridge, UK), HPI (Burlington, MA, USA), and Millipore (Burlington, CA, USA), respectively. Antibodies against CD31 and LYVE-1 were obtained from BD Biosciences (San Jose, CA, USA) and Angiobio (San Diego, CA, USA), respectively. Alexa Fluor 488-, 568-, and 647-conjugated antirabbit IgG and Hoechst 33258 were obtained from Invitrogen (Carlsbad, CA, USA).

### Expression and purification of the PCNs

The TFG/TFMG vectors were constructed, expressed, and purified as previously reported [11]. Briefly, TFG/TFMG plasmids were transformed into *Escherichia coli* expression strain BL21 (DE3) and grown in LB medium containing 50 μg/ml kanamycin. Protein expression was induced with 0.1 M IPTG at 37 °C for 5 h, and the cells were lysed (lysis buffer: 50 mM Tris-HCl pH 6.8, 100 mM NaCl, 1% Triton X-100, 1 mM PMSF, 1 mM DTT, protease inhibitor cocktail) and ultrasonicated. Binding was performed (urea binding buffer: 20 mM Tris-HCl pH 6.8, 300 mM NaCl, 10 mM imidazole, 8 M urea) at room temperature for 1 h. The sample was loaded onto nickel ion chelate affinity columns, washed (washing buffer: 20 mM Tris-HCl pH 6.8, 500 mM NaCl, 30 mM imidazole, 8 M urea), and eluted (elution buffer: 20 mM Tris-HCl pH 6.8, 100 mM NaCl, 300 mM imidazole, 8 M urea). Finally, sequential dialysis (dialysis buffer: 20 mM Tris-HCl pH 6.8, 100 mM NaCl, and sequentially diminishing concentration of urea) using a dialysis cassette (MWCO, 10 kDa; Thermo Scientific, Rockford, IL, USA) was conducted to obtain refolded proteins.

### Protein gel staining

SDS-PAGE gel staining assay was performed to evaluate the purity of TFG and TFMG following protein purification. First, the protein samples were separated by gel electrophoresis using a 10% SDS-PAGE gel, followed by washing and visualization of the protein bands by staining with a solution containing Coomassie brilliant blue R250 dye (0.125 g), glacial acetic acid (5 ml), methanol (25 ml), and water (20 ml), followed by destaining. Photographic images of the gels were captured to record the protein bands.

### Cell culture

LLC and EA.hy926 (human endothelial somatic cell hybrid) cells were obtained from the American Type Culture Collection (Manassas, VA, USA), and C3H/10T1/2 (clone 8) cells were obtained from the Korean Cell Line Bank (Seoul, South Korea). All of the cells were grown in DMEM (Hyclone, Logan, UT, USA) supplemented with 10% FBS (Hyclone, Logan, UT, USA) and 1% antibiotics (100 units/ml penicillin, 100 mg/ml streptomycin, and 0.25 mg/ml amphotericin B; Invitrogen, Carlsbad, CA, USA) at 37 °C in a humidified atmosphere containing 5% CO_2_ and 95% air.

### Mice

The mice used in this study consisted of 5- to 6-week-old adult male C57BL/6J mice (SLC, Japan) or *MMTV*-*PyMT* transgenic mice [20, 21] (Jackson Lab, Sacramento, CA, USA). All mice were housed in a specific pathogen-free facility with individual ventilation systems at Kyungpook National University. The animal handling and experimental procedures were conducted strictly according to the Guidelines for Care and Use of Laboratory Animals issued by the Institutional Ethical Animal Care Committee of Kyungpook National University (Protocol number: KNU 2014-0189 and KNU 2017-0145).

### Pharmacokinetic study

After intravenous injection of TRAP (10 nmol/kg) into the tail vein of mice, plasma samples (30 μl) were obtained at 0.5, 1, 1.5, and 2 h. Plasma samples (30 μl) were vigorously mixed with 100 μl acetonitrile and centrifuged at 16,000 g for 10 min. Aliquots (20 μl) of the supernatant were injected into an Agilent 6470 Triple Quadrupole mass spectrometer equipped with an Agilent infinity 1260 high-performance liquid chromatograph (Agilent, Wilmington, DE, USA). TRAP was monitored using multiple reaction modes at m/z 761.6 → 602.5 in the ionization mode with a collision energy of 35 eV. After intravenous injection with DyLight 680 NHS ester-conjugated TFMG (10 nmol/kg) into the tail vein of LLC allograft tumor-bearing mice, plasma samples (30 μl) were obtained at 0.5, 2, 4, 8, 24, and 48 h. The fluorescence of the TFMG in plasma samples was measured at excitation and emission wavelengths of 685 nm and 715 nm, respectively. The calibration standards for TRAP and TFMG in mouse plasma were linear in the range of 0.28–72 nM with a correlation coefficient of over 0.999. The pharmacokinetic parameters were calculated using the non-compartmental model in WinNonlin (version 5.1; Pharsights, Cary, NC, USA).

### Development of tumor models and treatment regimens

To generate a LLC allograft tumor model, suspensions of LLC cells (4 × 10^5^ cells in 200 μl of serum-free culture media) were implanted subcutaneously into the dorsal flank regions of 5- to 6-week-old male C57BL/6J mice. At the indicated time points following LLC cells inoculation, the mice were administered TFG (10 nmol/kg) or TFMG (10 nmol/kg), with or without cisplatin (3 mg/kg), or bevacizumab (5 mg/kg) by intravenous injection into the tail vein. Subsequently, tumor volumes were measured based on the formula: *V* = 0.5 × *A* × *B*^2^, where *A* represents the longest and *B* the shortest dimension. Eventually, the mice were anesthetized and tissues were harvested for further analyses at the completion of the study period.

Female *MMTV*-*PyMT* transgenic mice were employed as another model to observe tumor growth and metastasis. Twelve weeks following their birth, the volumes of every palpable tumor nodule were measured and added to determine the tumor burden for each mouse. Based on tumor burden, the mice were divided into three groups, each receiving TFG (10 nmol/kg), TFMG (10 nmol/kg), or vehicle (control) at the indicated time points. Tumor burdens after each week following the 12th week were measured up to the 15th week for each of the three groups. At the 15th week, the mice were anesthetized and tissues were harvested for further analyses.

### Immunohistochemical and histological analyses

For immunohistochemical (IHC) analysis, the harvested tumor or tissue samples were fixed in 3.7% formaldehyde (FA), dehydrated by serially incubating with 10% to 40% sucrose solution, and finally embedded in tissue freezing medium (Leica, Wetzlar, Germany). The frozen blocks were cut into 5 to 10 μm sample sections, which were blocked with 5% BSA in PBST (0.3% Triton X-100 in phosphate-buffered saline (PBS)) and incubated overnight at 4 °C with the appropriate primary antibody: anti-CD31 (BD Biosciences, CA, USA), anti-CD31 (Milipore, MA, USA), anti-α-SMA (Abcam, Cambridge, UK), antipimonidazole (Hypoxyprobe-1, HPI, MA, USA), anticytokeratin II (Millipore, MA, USA), anti-LYVE-1 (Angiobio, CA, USA), anti-Phospho-Tie-2 (R&D systems, MN, USA), anti-PAR1 (Abcam, Cambridge, UK), anti-PAR3 (Santa Cruz, CA, USA), anti-CD68 (Abcam, Cambridge, UK), anti-iNOS (Abcam, Cambridge, UK), anti-arginase1 (BD Biosciences, CA, USA), anti-CD4, PE (Invitrogen, CA, USA), or Alexa Fluor 647 anti-CD8a (BioLegend, CA, USA). After a few washes, the samples were incubated at RT for 1 h with the recommended fluorophore-tagged secondary antibody, their nuclei stained with Antifade Mounting Medium with DAPI (Vector Laboratories, Burlingame, CA) or Hoechst 33258 (Invitrogen, USA), and mounted with fluorescent mounting medium (Sigma, USA). The immunofluorescence images were acquired using a Leica TCS SP5 II confocal microscope (Leica, Wetzlar, Germany) or a Zeiss confocal microscope (Carl Zeiss, Germany). Pimonidazole hydrochloride (60 mg/kg; Hypoxyprobe-1, HPI, USA) was injected intraperitoneally 90 min before sacrifice of mice bearing tumors used for the investigation of hypoxic regions. Serial tissue sections (5 μm) were stained with H&E and observed under an Axio Imager A1 light microscope (Carl Zeiss, Germany).

### Terminal deoxynucleotidyl transferase-mediated dUTP nick-end green fluorescent labeling assay

Tumor tissues were fixed with 4% paraformaldehyde in PBS at 4 °C for 24 h and frozen sections were prepared. The terminal deoxynucleotidyl transferase-mediated dUTP nick-end green fluorescent labeling (TUNEL) assay was performed with sectioned tissues (5–10 μm thickness) according to the manufacturer’s instructions (TUNEL assay kit, Promega, Madison, WI, USA). Fluorescent images were observed by fluorescence microscopy (Axio Imager A1 microscope Carl Zeiss, Germany).

### In vivo vascular leakage and perfusion assays

On the day of the last PCN injection, a vascular perfusion assay was performed by injecting 100 μl of DyLight® 488-labeled *Lycopersicon esculentum* (tomato) lectin (1 mg/ml, Vector Lab) intravenously 30 min prior to sacrifice. To check for vascular leakage, 100 μl of FITC-dextran (25 mg/ml, 70 kDa, Sigma-Aldrich) was intravenously injected into the mice 30 min before sacrifice. After the mice were perfused with PBS and 4% paraformaldehyde (PFA), the FITC-dextran or tomato-lectin in the tumor tissues were observed under a Leica TCS SP5 II Dichroic/CS confocal microscope (Leica, Wetzlar, Germany). The fluorescence intensity was measured using ImageJ software.

### Conjugation of fluorophores to TFMG

TFMG was labeled with Dylight 680 NHS ester at a molar ratio of 1:10. Briefly, TFMG (1 mg) was dissolved in PBS (1.5 ml) and Dylight 680 NHS ester (1 mg) was dissolved in DMSO (0.1 ml). TFMG and fluorescent dye were incubated for 2 h at room temperature. The reaction product was passed through a 0.2-μm filter unit, and the unreacted dye was separated on a PD midiTrap™ G-25 (GE Healthcare, UK) column that had been pre-equilibrated with PBS and 2 mM sodium azide. This process yielded 300 μM of TFMG with a ratio of dye per protein greater than 1.5.

### In vivo imaging for biodistribution

The animal study was conducted using the same protocol described above. A total of 8 C57BL/6J mice were randomized and grouped. Two mice were allocated to receive non-labeled TFMG treatment (control). For the TFMG-treated group, 6 mice were divided into 2 time points (*n* = 3): 3 and 6 h. TFMG was diluted in saline and administered intravenously once at an equivalent dose of 5.48 μg/mouse via the tail vein. The animals were imaged by IVIS® Spectrum CT (Perkin Elmer, Waltham, MA).

### Gene silencing and quantitative real-time polymerase chain reaction

EA.hy926 and C3H/10T1/2 cells were transfected with 10 to 200 nM of siRNA for *PAR*-*1*, *PAR*-*2*, *PAR*-*3*, *Tie2*, *G*_*α13*_, *G*_*αi*_, or *G*_*αq*_ (Bioneer, Daejeon, South Korea) using Lipofecatmine RNAiMAX. Silencing of the genes was confirmed by quantitative real-time polymerase chain reaction (qRT-PCR) using the primers shown in Table S2. After total RNA was extracted using a Trizol RNA extraction kit (Invitrogen, California, USA), cDNA was reverse-transcribed using ReverTra Ace® qPCR RT Master Mix (TOYOBO, Osaka, Japan). Quantitative real-time PCR was performed with SsoAdvanced Universal SYBR® Green Supermix (Bio-rad, Hecules, CA, USA) (Bio-rad C1000 Thermocycler). Relative expression to β-actin was calculated by the ΔΔCt method (Livak and Schmittgen 2001).

### In vitro cell co-cultures

Endothelial cell/cancer cell (EC) co-cultures, pericyte/cancer cell (PC) co-cultures, and pericyte/endothelial cell/cancer cell (PEC) co-cultures were established using EA.hy926, LLC, and C3H/10T1/2 cells. For the EC co-cultures, LLC cells (2.5 × 10^5^ cells/well) were first seeded in 12-well plates and allowed to adhere for 4 h, after which 12-well Transwell polycarbonate membrane inserts (3 μm; Corning, NY, USA) were placed above each well. EA.hy926 cells (5 × 10^5^ cells/well) were seeded over the Transwell membrane inserts and incubated at 37 °C for 48 h. For PC co-cultures, LLC (2.5 × 10^5^ cells/ well) cells were seeded onto the 12-well plate surface, while C3H/10T1/2 cells (2.5 × 10^5^ cells/well) were seeded above the Transwell membrane inserts. For the PEC co-cultures, LLC (2.5 × 10^5^ cells/ well) cells were seeded onto the 12-well plate surface, and C3H/10T1/2 cells (2.5 × 10^5^ cells/well) were added to the lower surface of the Transwell membrane inserts and was followed by the addition of EA.hy926 cells (5 × 10^5^ cells per well) onto the upper surface of the Transwell membrane inserts. The other procedures remained the same.

### In vitro cell permeability analysis

EC, PC, and PEC co-cultures were used to conduct permeability assays. The EC, PC, and PEC co-cultures were treated with TFG or TFMG and incubated at 37 °C for 24 h. Transwell membrane inserts were washed with PBS and transferred to fresh 12-well plates containing 500 μl of serum-free DMEM per well. FITC-dextran (150 μl; 50 μg/ml; MW = 3 kDa; Sigma-Aldrich, MO, USA) was added to each of the Transwell membrane inserts, and leakage across the Transwell membrane was determined using a fluorescence plate reader (λ_ex_, 485 nm; λ_em_, 535 nm).

### Immunoprecipitation

EA.hy926 cells (1 × 10^6^) were seeded in 100 mm cell culture plates, incubated overnight at 37 °C, and treated with 100 nM TFG or TFMG for 24 h. The cells were then washed with PBS, detached, and lysed with RIPA buffer solution. An appropriate amount of protein was mixed with anti-PAR-3 antibody and incubated overnight at 4 °C under constant rotation. Magnetic beads were added and the mixture was rotated at 4 °C for 4 h to permit binding. After washing and eluting, immunoblots were recorded to visualize the co-immunoprecipitated proteins.

### Western blot analysis

EA.hy926 cells were seeded into 6-well plates, incubated overnight at 37 °C, and treated with 100 nM TFG or TFMG for a suitable duration. The cells were then washed with PBS, detached, and lysed with RIPA buffer solution. Equal amounts of proteins were separated using SDS-PAGE gels and transferred to PVDF membranes. After blocking, the membranes were incubated with antibodies against p-Tie2 (1:1000), Tie2 (1:200), p-Akt (1:1000), Akt (1:1000), FoxO1 (1:1000), p-FoxO3a (1:1000), FoxO3a (1:1000), ROCK-1 (1:1000), ZO-1 (1:1000), or β-actin (1:5000) at 4 °C for 12 h. Membranes were washed three times for 10 min and incubated with a 1:3000 dilution of anti-mouse, anti-goat, or antirabbit antibodies for 2 h. The Western blots were visualized by chemiluminescent detection.

### Immunofluorescence

EA.hy926 cells (2 × 10^5^ cells/well) were seeded onto glass coverslips in 4-well plates, incubated overnight at 37 °C, and treated with 100 nM TFMG for 24 h. The cells were then washed with PBS, fixed with 3.7% formaldehyde (Sigma-Aldrich, St Louis, MO, USA), and permeabilized with 0.1% Triton X-100 (Amresco, Solon, OH, USA). After 1 h of blocking with 3% BSA, the cells were incubated overnight at 4 °C with the required primary antibody solution in 0.1% BSA. After washing with PBS the following day, the cells were incubated at room temperature for 1 h with the appropriate fluorescence-tagged secondary antibody solution in 0.1% BSA. The coverslips were mounted onto glass slides using mounting gel and the images were visualized and recorded using a Leica TCS SP5 II confocal microscope (Leica, Wetzlar, Germany).

### Statistical analysis

Statistical analysis was carried out using Graph Pad Prism 7.0. Significant differences for individual pairs of means were determined using Student’s *t* test and for lager datasets comparing more than 3 groups, one-way ANOVA was used followed by post hoc Dunnett’s multiple comparison test where appropriate to analyze the level of significance, with *p* values < 0.05 considered statistically significant. Two-way ANOVA was used when there is more than one independent variable and multiple observation for the mean differences between groups. All the data were expressed as means ± standard deviation (SD) of at least 3 replicates. For the analysis of synergism of combination therapy, the Bliss independence model was utilized [22]. The expected response tumor volume (*V*_Exp_) for the combination groups was defined by the formula: *V*_Exp_ = (*V*_1_ × *V*_2_)/*V*_C_, where *V*_1_ and *V*_2_ are the mean tumor volumes in the single-treatment group and *V*_C_ is the mean volume in the control group. The tumor volume change from the baseline of control group was defined as Δ*V* = *V*_C_ − *V*_0_ (initial volume). Then, the range around the *V*_Exp_ was calculated, via upper (*U*) and lower (*L*) limits, as follows: *V*_Exp_*U* = *V*_Exp_ + 0.15 × Δ*V* and *V*_Exp_*L* = *V*_Exp_ − 0.15 × Δ*V*. If *V*_Obs_ is less than *V*_Exp_*L*, it indicates a synergistic effect, and if *V*_Obs_ is in-between *V*_Exp_L and V_Exp_U, it denotes an additive effect. *V*_Obs_: the observed mean tumor volume in the combination groups.

## Results

### TFMG exhibits a high pharmacokinetic stability in vivo

We constructed short ferritin (sFn) by deleting the short helix E and loop from the human ferritin light chain, and produced the TRAP-ferritin-PC-Gla (TFG) protein by inserting the EPCR ligand (PC-Gla domain) at the C-terminus and PAR-1 activator (TRAP peptide) at the N-terminus to the genetically engineered sFn (Fig. S1A-S1B) [11]. A matrix metalloproteinase (MMP)-2 cleavage site in-between the sFn and PC-Gla domains was alternatively inserted to enable the release of PC-Gmla from the nanocages at MMP-2-activating sites, called TFMG (TRAP-sFn-MMP-2-PC-Gla). Thus, the potential interference of PC-Gla with remaining TRAP-sFn during simultaneous double binding to respective receptors was avoided. We reproducibly generated highly pure PCNs showing strong TFG and TFMG bands, corresponding to their molecular weights of approximately 27 kDa and 27.5 kDa, respectively, whereas no secondary bands were observed throughout the gel other than the specific TFG and TFMG bands, indicating a high purity (Figure S1C). We used the same constructs produced in our previous study in which we already determined particle size by dynamic light scattering analyses as well as TEM (Fig. S1D) [11]. The mean outer diameters of TFG and TFMG were 17.0 and 18.5 nm, respectively (Fig. S1E), which were increased compared with the size of wild-type ferritin (6.9 nm) [23].

**Fig. 1:**
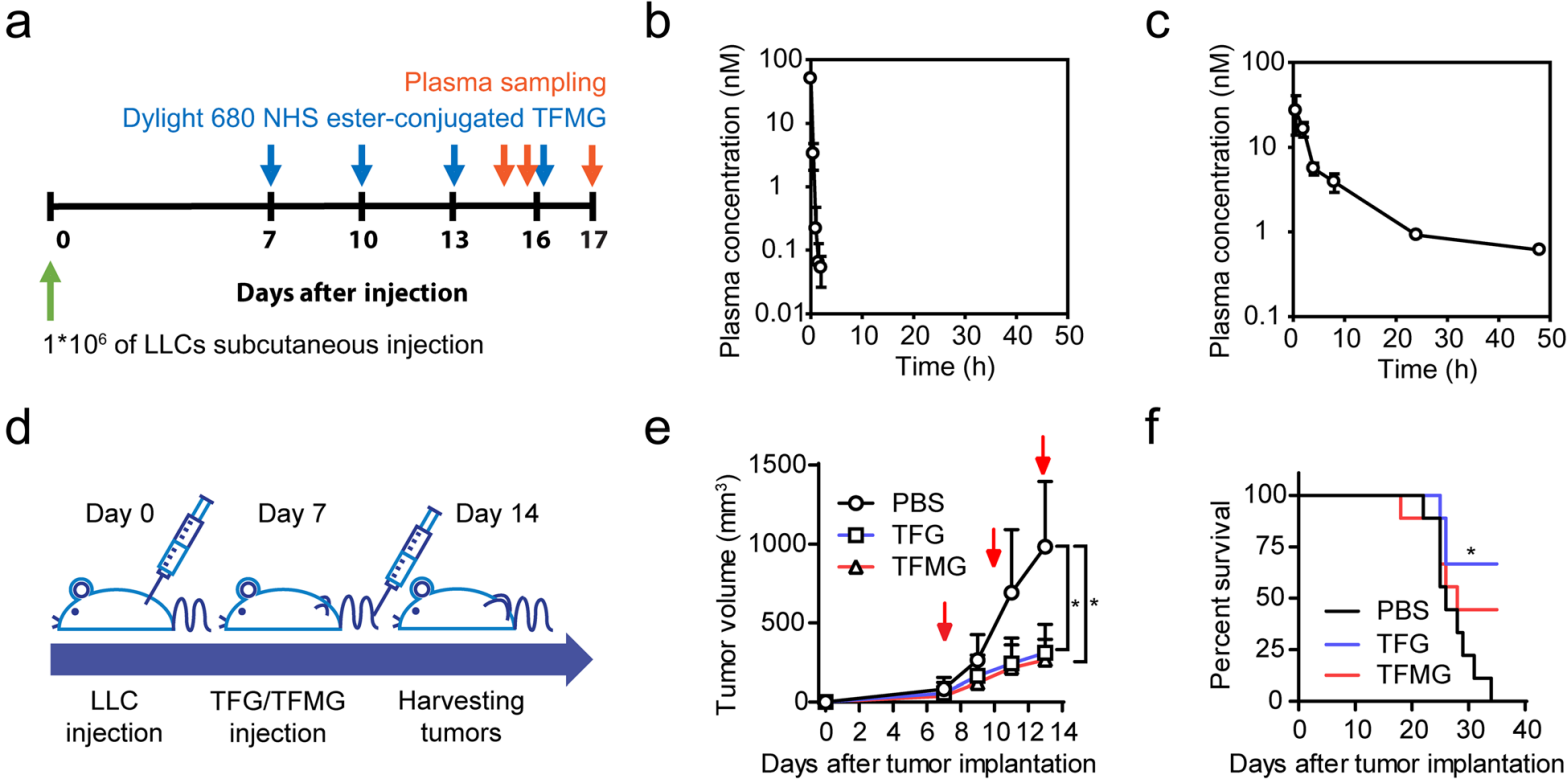
TGMG exhibits high pharmacokinetic stability and PCNs suppress tumor growth in vivo. **a** Schematic diagram depicting the schedule of TRAF or TFMG treatment and plasma sampling in LLC allograft models. LLC tumor-bearing mice, following LLC cell inoculation, were administered TRAF peptide (10 nmol/kg), TFMG (10 nmol/kg), or vehicle control (PBS) subcutaneously. The blue arrowheads represent the point of TRAF or TFMG administration and the red arrowheads represent the point of plasma sampling. **b**, **c** Plasma concentrations of TRAP (**b**) and TFMG (**c**) in LLC tumor-bearing mice following intravenous injection of TRAP (10 nmol/kg) or TFMG (10 nmol/kg), respectively (*n* = 3). **d** Schematic diagram depicting the development of the LLC allograft models and treatment schedule. LLC tumor-bearing mice, following LLC cell inoculation, were administered TFG (10 nmol/kg), TFMG (10 nmol/kg), or vehicle control (PBS) intravenously on days 7, 10, and 13. The tumors were sampled on day 14. **e** Pattern of tumor growth in LLC tumor-bearing mice treated with TFG, TFMG, or vehicle control (PBS). Tumor volumes were measured on days 7, 9, 11, and 13. The red arrowheads represent the point of TFG/TFMG administration (*n* ≥ 9). **f** Survival curves of the LLC tumor-bearing mice. Data information: Data are presented as the mean ± SD. Significant enrichment: *P < 0.0001 (two-way ANOVA) in (**e**); *P < 0.01 (Mantel-Cox test) in (**f**)

We investigated the plasma stability of the TRAP domain and the pharmacokinetic properties of TFMG in LLC tumor-bearing mice (Fig. 1a). The TRAP peptide alone was unstable in the mouse plasma with a half-life of 9.5 min (Fig. 1b). These results suggest that when administered in vivo, TRAP itself could not stably initiate pharmacological activity. However, TFMG showed a much longer half-life (13.83 ± 1.08 h) and plasma TFMG concentration was maintained over 0.6 nM for 48 h following intravenous injection of TFMG (10 nmol/kg) (Fig. 1c). The pharmacokinetic parameters calculated from the plasma concentration profile of TFMG are shown in Table S1. Collectively, the pharmacokinetic results and plasma stability for TFMG suggest an advantage of TFMG nanoparticles as anticancer therapeutics compared with the use of TRAP peptide alone.

### PCNs inhibit tumor growth in an LLC tumor model

To investigate the effects of PCNs on tumor growth, we used an LLC allograft tumor model, in which TFG (10 nmol/kg), TFMG (10 nmol/kg), or vehicle (control) was administered at days 7, 10, and 13 following LLC cell inoculation (Fig. 1d). We found that both TFG and TFMG significantly suppressed tumor growth (Fig. 1e) and prolonged survival (Fig. 1f) compared with the controls. In addition, the PCNs exhibited good delivery efficacy to the tumor mass and various organs (Fig. S2A), whereas they did not induce hemorrhage in the internal organs or the tumor masses (Fig. S2B and S2C). Thus, we examined the effects of TFG/TFMG on apoptosis in the major organs by performing TUNEL assays (Fig. S2D). Apoptotic cells at the kidney and heart were not visible in both vehicle control and treatment group, but reduced apoptotic cells were observed at the liver, lung, and spleen at TFG/TFMG-treated group compared to the control group. To determine the maximal effects of the PCNs, we examined various administration routes including intravenous, subcutaneous, and intramuscular routes in the LLC allograft tumor model (Fig. S2E). We found that TFMG administration by an intravenous route exhibited higher antitumor effects compared with other administration routes without causing reduced body weight or tissue damage (Fig. S2F-S2H). Based on these results, we administrated TFMG by an intravenous route in subsequent mouse model studies. These results suggest that TFG and TFMG have antitumor effects with good delivery efficacy in vivo.

### PCNs inhibit tumor metastasis in mouse models

**Fig. 2:**
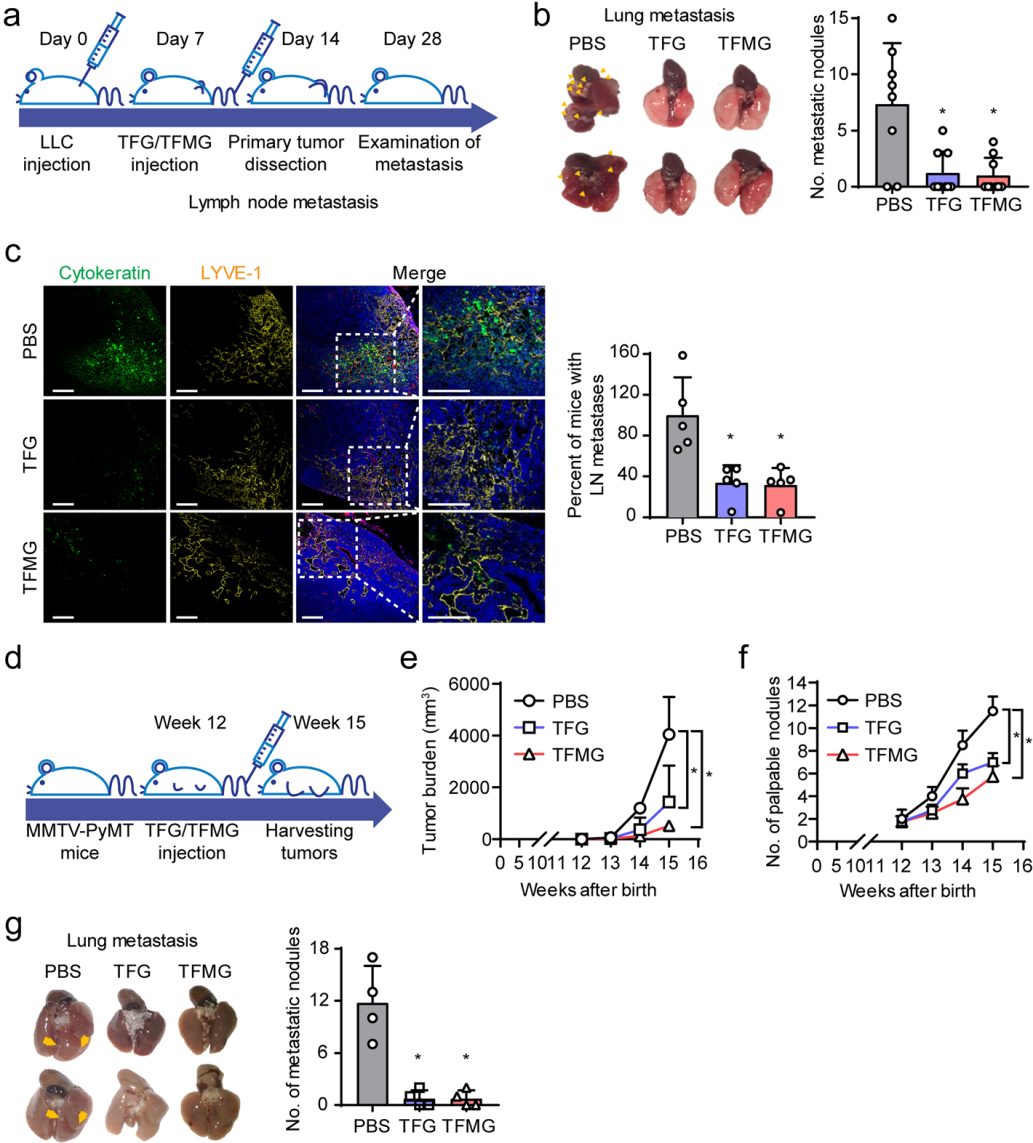
TFG and TFMG suppresses metastasis in mouse models. **a** Schematic diagram depicting the generation of metastatic tumor models and treatment schedule. LLC tumor-bearing mice, following LLC cell inoculation, were administered, intravenously, TFG (10 nmol/kg), TFMG (10 nmol/kg), or vehicle control (PBS) on days 7, 10, and 13, and the primary tumors were dissected on day 14. Inguinal lymph nodes (LNs) and lungs were sampled on day 28. **b** Representative photographic images of metastatic lungs resected from mice from the control, TFG, or TFMG groups. Graphical representation of the observed metastatic nodules in each group (*n* ≥ 8). **c** Confocal images of sections of inguinal LNs showing cytokeratin+ tumor metastatic areas. Sections of inguinal LNs from vehicle control (PBS), TFG, or TFMG-treated mice were stained with cytokeratin (green, epithelial cells), LYVE-1 (yellow, lymphatic vessels), and Hoechst 33258 (blue, nuclei) (*n* = 5). Scale bars: 100 μm. Quantitation (**c**) was done using ImageJ software. **d** Schematic diagram depicting the treatment schedule for MMTV-PyMT mice. Tumor growth was analyzed weekly in the MMTV-PyMT transgenic mouse models from week 12 and onwards following birth. The mice were treated with TFG (10 nmol/kg), TFMG (10 nmol/kg), or vehicle control (PBS) every 3 days until the tumors were sampled and analyzed at week 15. **e** Pattern of increase in tumor burden in the vehicle control (PBS), TFG-, or TFMG-treated MMTV-PyMT mice. Tumor burden was calculated as the sum of the volumes of all tumor masses (*n* = 4). **f** Pattern of increase in the number of palpable tumor nodules in the MMTV-PyMT mice (*n* = 4). **g** Photographic images of representative metastatic lungs resected from the MMTV-PyMT mice at week 15. The yellow arrowheads indicate metastatic nodules in the lungs of the respective groups (*n* = 4). Data are presented as the mean ± SD. Data information: Data are presented as the mean ± SD. Statistical analysis was done using two-way ANOVA for growth curves or a student *t* test for No. of metastatic nodules. *P < 0.05 (**b**, **c**), *P < 0.0001 (**e, f**), *P < 0.001 (**g**).

We also examined the effects of the PCNs on tumor metastasis in an LLC allograft tumor model. The PCNs were administered every third day from day 7 to 28 (8 times) following LLC cell inoculation. The tumors were dissected from the mice on day 14 after inoculation and examined for lung metastasis on day 28 (Fig. 2a). Interestingly, we found that TFG and TFMG significantly reduced tumor metastasis and decreased the number of metastatic nodules in the lungs (Fig. 2b). Additionally, we examined tumor metastasis using cytokeratin (epithelial cells marker) and LYVE-1 (lymphatic vessels marker) in cytokeratin+ tumor cells and found that TFG and TFMG significantly reduced metastasis to inguinal lymph nodes (Fig. 2c).

To further elucidate the anti-metastatic potential of the PCNs, we employed another tumor model, the *MMTV*-*PyMT* spontaneous breast cancer model, which spontaneously develops metastatic breast cancer. *MMTV*-*PyMT* mice were treated with TFG (10 nmol/kg), TFMG (10 nmol/kg), or vehicle every 3 days from week 12 following their birth up to week 14. Tumor size and the number of palpable tumor nodules were assessed at week 15 (Fig. 2d). We found that TFG and TFMG significantly reduced tumor burden (Fig. 2e) and decreased the number of tumor nodules (Fig. 2f) compared with control mice, respectively. In addition, we also found that TFG and TFMG significantly decreased metastatic nodules in the lungs (Fig. 2g). These results indicate that TFG and TFMG exhibit anti-metastatic effects in tumor implants and conventional transgenic tumor mouse models.

### PCNs induce normalization of the tumor vasculature in mouse models

**Fig. 3:**
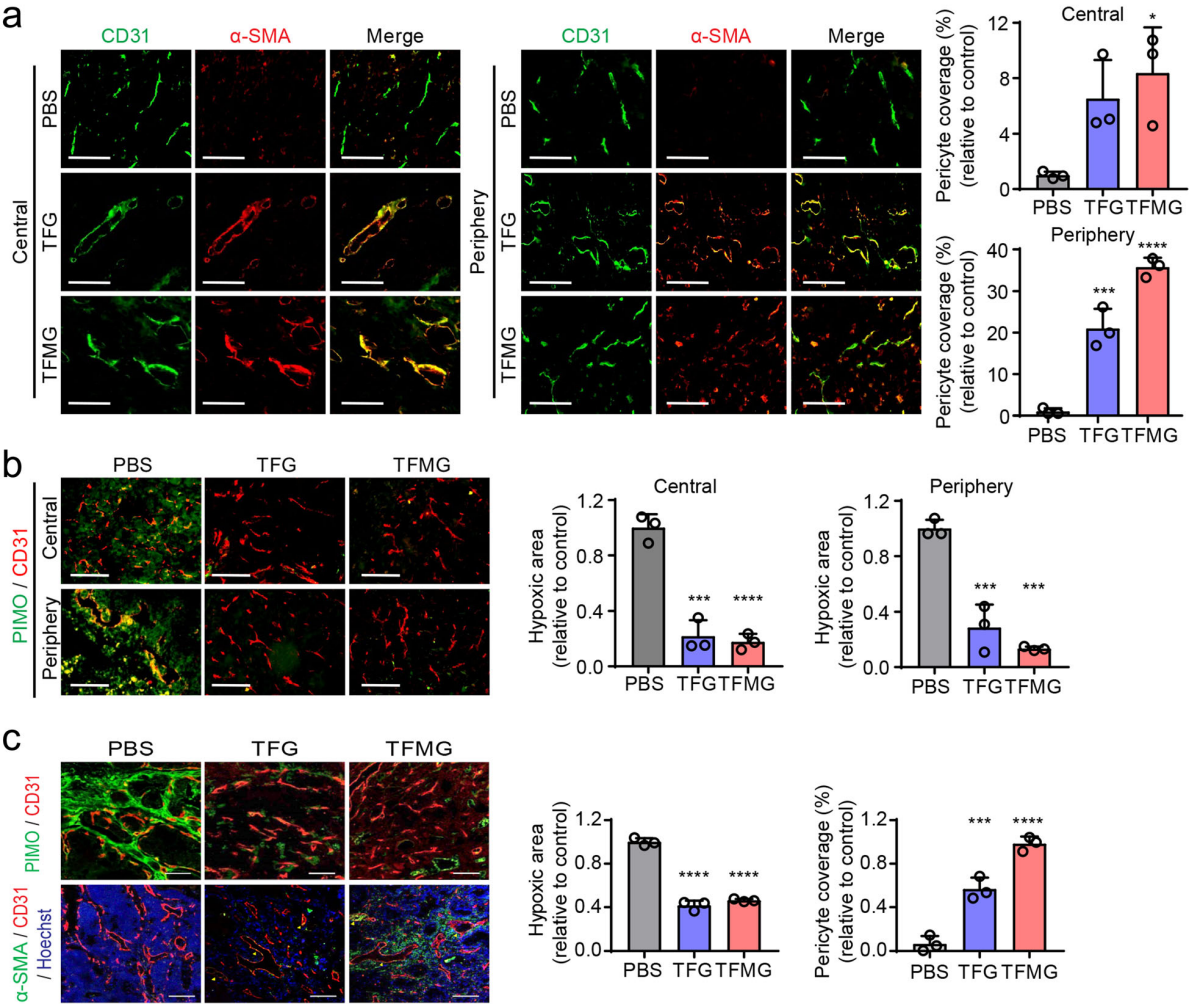
TFG and TFMG-induced tumor vascular normalization. **a** Confocal images of tumor sections showing α-SMA+ pericyte coverage on tumor vessels in the central and peripheral regions of allograft tumors from the LLC tumor model. Frozen tumor sections were stained with CD31 (green, blood vessels) and α-SMA (red, pericyte). Scale bars: 100 μm. Right graphs show the quantitation of fluorescent images by ImageJ software (*n* ≥ 3). **b** Confocal images of tumor sections showing hypoxic regions in the center and periphery of the LLC tumors stained with Hypoxicprobe™ (green, PIMO, hypoxic areas) and CD31 (red, blood vessels). Scale bars: 100 μm. Right graphs show the quantitation of fluorescent images by ImageJ software (*n* ≥ 3). **c** Confocal images of tumor sections showing hypoxic regions in the center of the tumor from the MMTV-PyMT mice stained with Hypoxicprobe™ (green, PIMO, hypoxic areas) and CD31 (red, blood vessels). Confocal images of tumor sections showing α-SMA+ pericyte coverage on tumor vessels. Frozen tumor sections were stained with α-SMA (green, pericyte), CD31 (red, blood vessels), and Hoechst 33258 (blue). Scale bars: 100 μm (*n* = 3). The quantitation in (**a**–**c**) were conducted using ImageJ software. Data information: Data are presented as the mean ± SD. Significant enrichment: *P < 0.05; ***P < 0.001; ****P < 0.0001 (Sidak’s multiple comparisons test)

Since previous studies have suggested that tumor vascular normalization is an alternative therapeutic strategy for malignant tumors by restoring the balance between pro- and antiangiogenic signaling [24], we examined the effects of the PCNs on remodeling the tumor vasculature in the mouse models. To determine PCN-mediated structural and functional vascular changes in the tumor core, we evaluated the pericyte coverage and hypoxic regions of the allograft tumor from the LLC tumor model. We found a significant increase of α-SMA^+^ pericyte coverage both in the central and peripheral tumoral regions (Fig. 3a). There was also a significant decrease of hypoxic regions (Fig. 3b, PIMO) following TFG or TFMG treatment compared with the controls. In addition, we found that TFG and TFMG significantly reduced the hypoxic regions in the tumor core while enhancing the pericyte coverage of blood vessels compared with controls in the *MMTV*-*PyMT* mouse model (Fig. 3c). These results suggest that the PCNs increase the normalization of abnormal tumor vasculature and decrease hypoxic areas within tumors, leading to inhibition of tumor growth.

### PCNs enhance the reconstitution of the tumor immune microenvironment

**Fig. 4:**
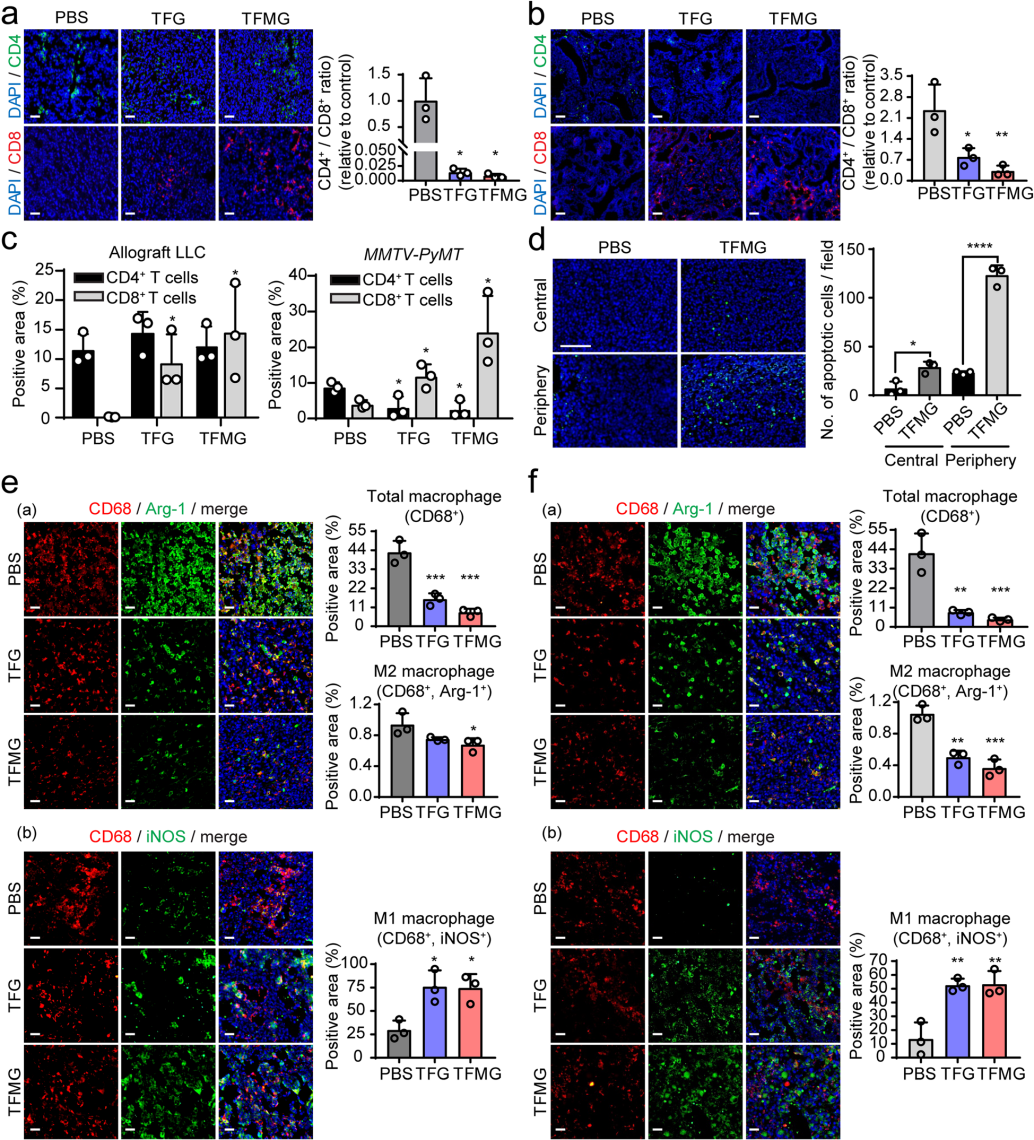
TFG and TFMG enhanced immune responses in LLC tumors and spontaneous MMTV-PyMT breast tumors. **a**, **b** Fluorescence microscopic images of tumor sections harvested from the LLC tumor-bearing mice (**a**) and from the MMTV-PyMT tumor-bearing mice (**b**) to illustrate CD4^+^ and CD8^+^ T lymphocytes. Frozen tumor sections from vehicle (PBS), TFG, or TFMG-treated groups were immunostained using PE-conjugated CD4 and Alexa Fluor 647-conjugated CD8a antibodies (*n* = 3). The scale bars: 50 μm. **c** The percentages of CD4^+^ T cells and CD8^+^ T cells population in the mouse tumor model with/without TFG or TFMG treatment (*n* = 3 per group). *P < 0.05 vs. the vehicle control (PBS). The data are presented as the mean ± SD. **d** TUNEL assay was performed with LLC and MMTV-PyMT tumor tissues in central and peripheral regions of the tissues, quantified for positive fluorescent cells, and graphed. DAPI staining for nucleus was performed (*n* = 3). ****P < 0.0001, *P < 0.05. The scale bars: 50 μM. **e**, **f** Macrophage infiltration into the LLC tumor (**e**) and into the MMTV-PyMT tumor (**f**) were evaluated. The number of infiltrating macrophages was determined by counting CD68^+^/arginase-1 (Arg-1)+ M2 macrophages (a) or CD68^+^/induced nitric oxide synthase (iNOS)+ M1 macrophages (b) in the tumor area. Quantitation of the CD68^+^, iNOS^+^, and Arg-1^+^ areas in three random microscopic fields in mice (*n* = 3) per group was performed using Image J software. Scale bars: 50 μm. Data information: Data are presented as the mean ± SD. Significant enrichment: *P < 0.05, **P < 0.01; ***P < 0.001; (Sidak’s multiple comparisons test)

Abnormal vasculature creates a hypoxic microenvironment that polarizes inflammatory cells toward immune suppression [24]. Therefore, to elucidate the underlying tumor microenvironmental changes through vascular normalization by PCNs, we examined whether PCNs induced changes in immune cell activity within tumor tissues. We found that the positive area stained with CD8 was significantly upregulated with minimal changes of the positive area stained with CD4 by TFG or TFMG treatment. The ratio of CD4^+^/CD8^+^ was significantly decreased in TFG or TFMG-treated groups relative to the control in allograft LLC tumor model (Fig. 4a) as well as in the MMTV-PyMT spontaneous breast cancer model (Fig. 4b). These suggested that TFG or TFMG treatment induced significantly upregulation of cytotoxic CD8^+^ T lymphocytes with minimal alteration of CD4^+^ T lymphocytes in the animal models (Fig. 4c). To check for functional cytotoxic T cell activity, we analyzed apoptosis by the TUNEL assay in tumor tissues. We found that TFMG treatment significantly increased apoptosis both in the central and peripheral regions of the tumor tissues compared with controls (Fig. 4d). Moreover, the population of CD68^+^/Arg-1^+^ M2-like macrophages was significantly decreased in LLC tumor tissues following TFG and TFMG treatment compared with controls (Fig. 4e(a)). In contrast, the population of CD68^+^iNOS^+^ M1-like macrophages was increased by TFG and TFMG in tumor tissues (Fig. 4e(b)). Similar results were observed in the *MMTV-PyMT* spontaneous breast cancer model (Fig. 4f(a and b)). Decreased M2-like macrophages were observed, whereas increased M1-like macrophages and CD8^+^ T lymphocytes were evident in tumor tissue. M1-like macrophages tend to normalize tumor vasculature, whereas M2-like macrophages take part in the formation of abnormal blood vessels [25, 26]. In addition, M1-like macrophages stimulate naïve T cells to elicit a Th1/cytotoxic response, while M2-like macrophages induce T cell responses without antitumor activity [27, 28]. Therefore, M1-like cells could suppress tumor growth, whereas M2-like cells promote it [27, 29–31]. PCN increased M1-like macrophages and diminished M2-like macrophages, as well as CD8^+^ T lymphocytes in tumor tissue, suggesting PCN might induce normalization of tumor vasculature through enhancing the reprogramming of macrophages.

### Reduction of in vitro endothelial permeability by the PCNs requires endothelial PAR-1/PAR-3 heterodimerization

**Fig. 5:**
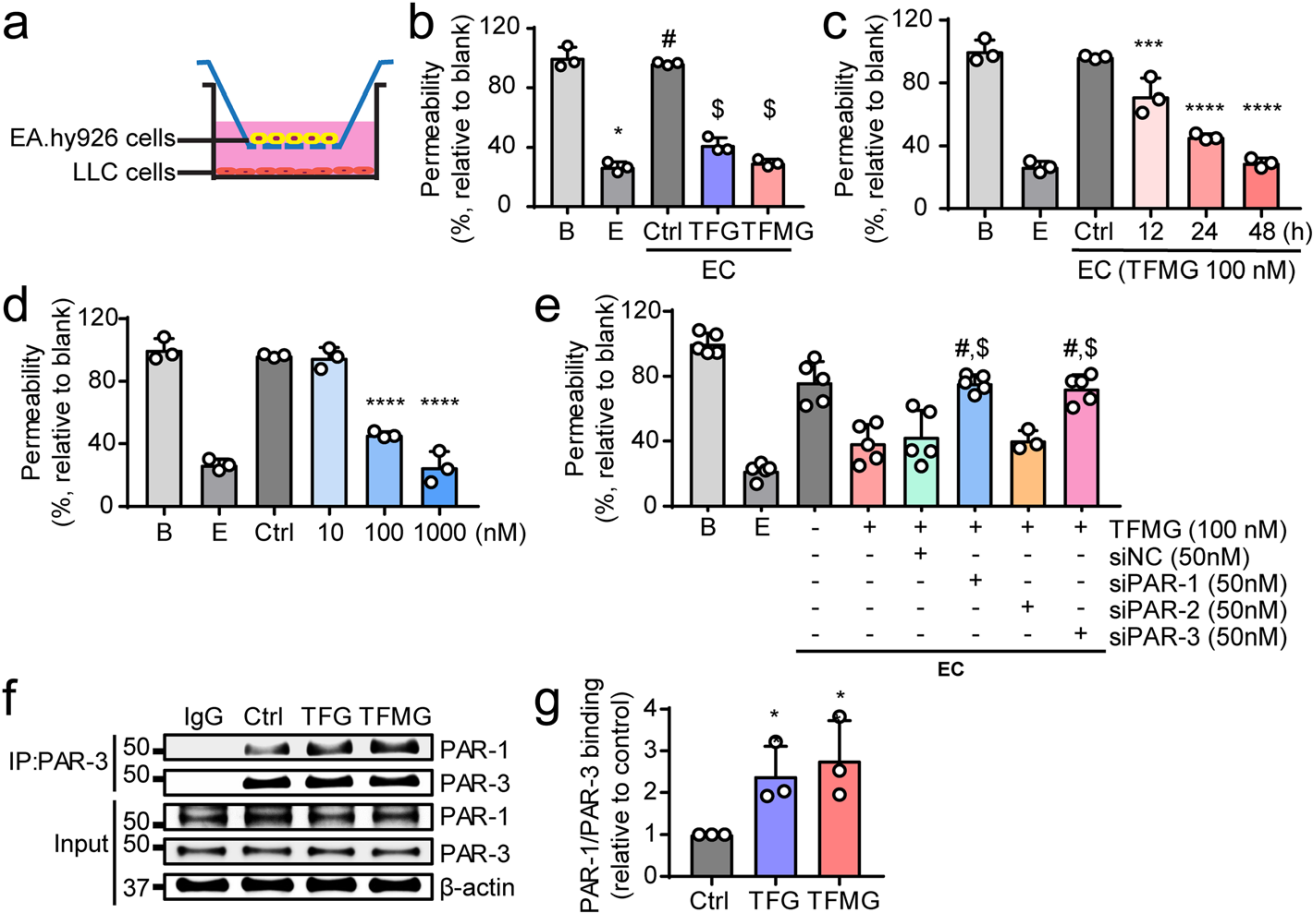
Endothelial PAR-3 is required for TFMG-induced normalization of vasculature. **a** Schematic diagram of the endothelial cell/cancer cell (EC) co-culture system. EA.hy926 cells (5 × 10^5^) were co-cultured with LLC cells (2.5 × 10^5^) in Transwell plates (pore size, 3 μm). **b** Permeability assay of TFG and TFMG using EC co-cultures. EC co-cultures were treated with TFG (100 nM) or TFMG (100 nM) for 48 h, and the permeability (%) of FITC-dextran was measured relative to untreated control co-cultures. B, Blank (no EA.hy926, no LLC); E, endothelial cell only (EA.hy926 seeded onto the Transwell membrane, no LLC); *P < 0.0001 vs. B; ^#^*P* < 0.0001 vs. E; ^$^*P* < 0.0001 vs. control (*n* = 3). **c, d** Permeability assay of TFMG using EC co-cultures to demonstrate the time- and dose-dependent effects. EC co-cultures were treated with 100 nM TFMG for 12, 24, or 48 h (**c**) and with 10, 100, and 1000 nM TFMG for 24 h (**d**), and the permeability (%) of FITC-dextran was measured relative to untreated control co-cultures (*n* = 3). **e** Effect of siRNA-mediated endothelial PAR-1, PAR-2, and PAR-3 silencing on TFMG-induced reduction of in vitro permeability. EC co-cultures, with or without siRNA-mediated silencing of endothelial PAR-1, PAR-2, or PAR-3, were treated with 100 nM TFMG for 24 h, and the permeability (%) of FITC-dextran was measured relative to untreated control co-cultures. *P < 0.0001 vs. untreated control EC co-culture; ^#^*P* < 0.001 vs. TFMG-treated EC co-culture; ^$^*P* < 0.001 vs. TFMG-treated EC co-culture with siNC (negative control siRNA) silencing; ns, no significant difference vs. TFMG-treated EC-cultures with or without siNC silencing (*n* = 5). **f, g** Co-immunoprecipitation assay of PAR-1 and PAR-3 following TFG or TFMG treatment. EA.hy926 cells were treated with TFG or TFMG (100 nM) for 24 h. The PAR-1/PAR-3 interaction was investigated by immunoprecipitation with anti-PAR3 antibody followed by western blot analysis using anti-PAR-1 antibody in TFG/TFMG-treated or untreated control cells (**f**). Relative binding of PAR-1/PAR-3 after TFG or TFMG treatment was quantitated using GelQuant software (**g**). Data information: Data are presented as the mean ± SD. Significant enrichment: *P < 0.05; ***P < 0.001; ****P < 0.0001 (**c**, **d**, **f**, **g**) (Sidak’s multiple comparisons test)

To investigate the molecular mechanism of PCN-induced vascular normalization, we established an in vitro EC co-culture system with LLC cells using Transwell plates (Fig. 5a). The EC co-cultures with TFG and TFMG significantly reduced vascular permeability in vitro (Fig. 5b) in both a time- and dose-dependent manner (Fig. 5c, d), confirming the in vivo animal results. PAR-1 signaling in endothelial cells has been known to involve activation of other protease-activated receptors, such as PAR-2 and PAR-3, by receptor heterodimerization [32–35]. Hence, we examined the involvement of PAR-2 and PAR-3 in the reduction of endothelial permeability induced by the PCNs. We performed an siRNA-mediated knockdown of PAR-1, PAR-2, and PAR-3, followed by measurement of the effects on TFMG-induced permeability abatement. The siPAR-1, siPAR-2, and siPAR-3 siRNAs exhibited more than 90% efficiency in suppressing gene expression in the ECs at 48 h post-transfection (Fig. S3). The endothelial permeability was significantly enhanced by PAR-1 and PAR-3 knockdown in ECs. However, PAR-2 knockdown had no significant effect on the reduction of endothelial permeability induced by TFMG (Fig. 5e), suggesting that only PAR-1 and PAR-3 are involved in the anti-permeable effect of PCNs in ECs.

Notably, PAR-3 was shown to potentiate the PAR-1-mediated response by PAR-1/PAR-3 heterodimerization [32]. Therefore, to test the role of the PAR-1 interaction with PAR-3 in the PCN-mediated effect, we examined whether TFG and TFMG treatment increased the PAR-1/PAR-3 interaction. We performed a co-immunoprecipitation analysis of PAR-1 and PAR-3 following TFG or TFMG treatment and found that the PAR-1/PAR-3 interaction was significantly enhanced (Fig. 5f). We also observed increased binding of PAR-1/PAR-3 by TFG and TFMG treatment (Fig. 5g) compared with the control. Together, these results suggest that both TFG and TFMG induce vascular normalization through heterodimerization of PAR-1 and PAR-3, leading to PAR-1-mediated PAR-3 activation.

### Activation of endothelial Tie2 is crucial for TFMG-induced vascular normalization

**Fig. 6:**
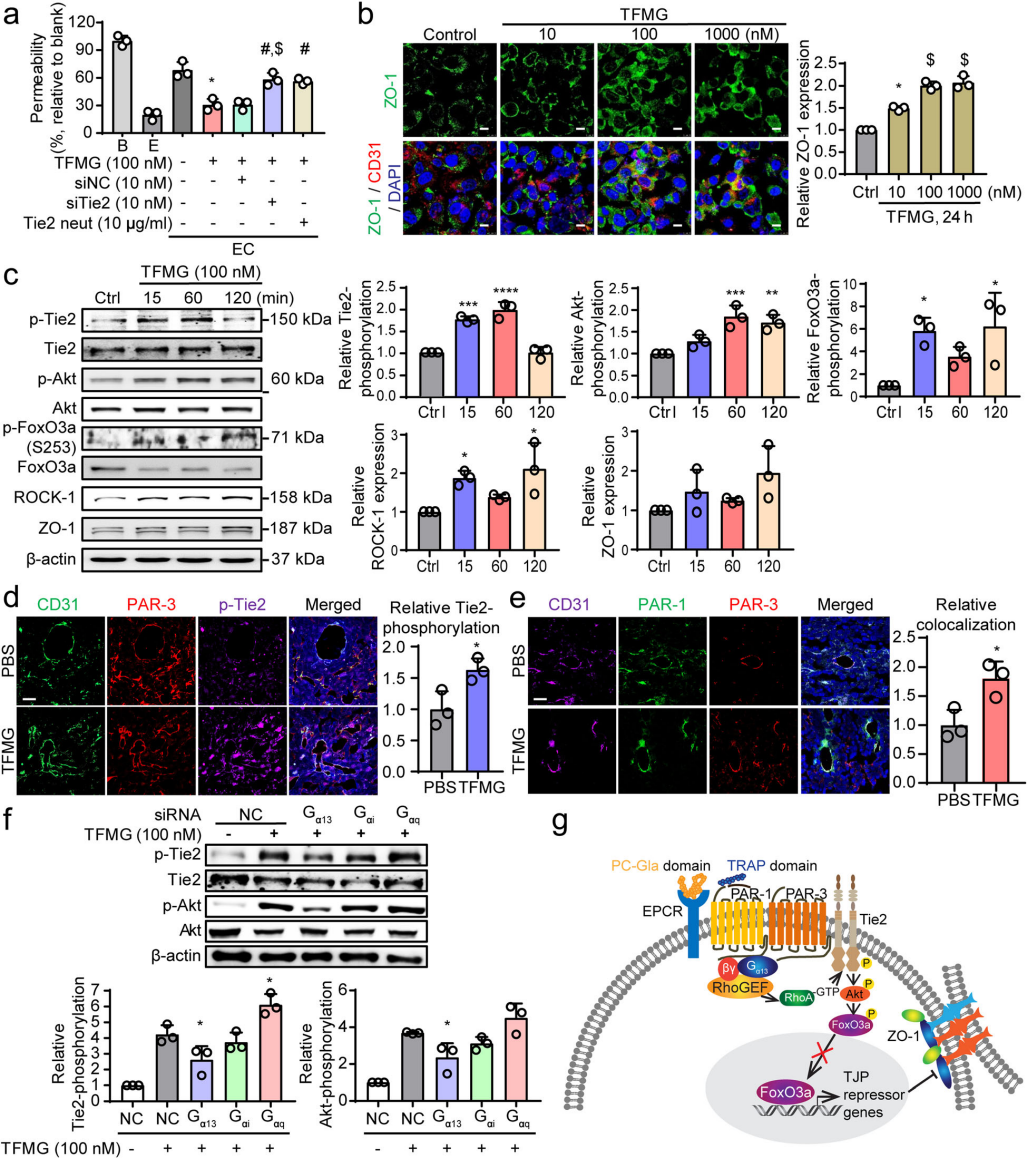
Endothelial Tie2 activation is essential for TFMG-induced vascular normalization. **a** EC co-cultures, with or without siRNA-mediated silencing of endothelial Tie2 or Tie2 neutralizing antibody-mediated blockage of Tie2, were treated with 100 nM TFMG for 24 h, and the permeability (%) of FITC-dextran was measured relative to untreated control co-cultures. *P < 0.0001 vs. untreated control EC co-culture (E); ^#^*P* < 0.01 vs. TFMG-treated EC co-culture; ^$^*P* < 0.001 vs. TFMG-treated EC co-culture with siNC silencing (*n* = 3). **b** EA.hy926 cells were treated with 10, 100, and 1000 nM of TFMG for 24 h, and immunofluorescent images were recorded to illustrate the level of ZO-1. (right panel) Quantitation of the relative ZO-1 level was performed. *P < 0.001 vs. Ctrl; ^$^*P* < 0.0001 vs. Ctrl (*n* = 3). **c** Western blot analyses to demonstrate the activation of Tie2 and Akt, along with the levels of ROCK-1 (Rho kinase), FoxO3a, and zonula occludens-1 (ZO-1) in endothelial cells following TFMG treatment. EA.hy926 cells were treated with 100 nM TFMG for 15, 60, and 120 min, and Western blot analysis was done to visualize the levels of phosphorylated Tie2 (p-Tie2), Tie2, p-Akt, Akt, ROCK-1, p-FoxO3a, FoxO3a, FoxO1, and ZO-1. **d**, **e** A frozen section of tumor tissues of each group was costained with anti-CD31/anti- PAR-3/anti-pTie2 antibodies (**d**) or anti-CD31/anti-PAR-1/anti-PAR-3 antibodies (**e**) and appropriate secondary fluorescent antibodies by IHC. Immunofluorescence was observed under the fluorescent microscope (× 400). Scale bar: 50 μm. **f** Ea.hy926 cells were transfected with negative control (NC), Gα13, Gαi, or Gαq siRNA (100 nM). Cells were harvested at 60 min after TFMG (100 nM) treatment and analyzed for activation of Tie2 signaling and Akt expression by western blot analysis. Quantitation in (**b–d**) were conducted using ImageJ software. **g** Proposed underlying mechanism of TFG and TFMG-induced vascular normalization in cancer. EPCR binding and PAR-1 activation by TFG/TFMG resulted in PAR-1/PAR-3 heterodimerization, which induced Gα13-mediated endothelial Tie2 activation, resulting finally in the induction of tight-junction proteins and vascular normalization. Data information: Data are presented as the mean ± SD. Significant enrichment: *P < 0.05; **P < 0.01; ***P < 0.001 (**c**, **d**) (Sidak’s multiple comparisons test)

The tyrosine-protein kinase receptor, Tie2, expressed primarily in endothelial cells, has recently been identified as a crucial target for vascular normalization in cancer [21, 36]. Tie2 activation has been implicated in noncanonical PAR-3 activation-mediated stabilization of vascular integrity [37]. To investigate whether Tie2 has a role in TFG/TFMG-induced vascular normalization, we examined the effects of siRNA-mediated endothelial Tie2 silencing and antibody-mediated blockage of endothelial Tie2 receptors on in vitro vascular permeability using EC co-cultures. As measured by qRT-PCR, Tie2 siRNA exhibited more than 90% efficacy in suppressing gene expression at 48 h post-transfection (Fig. S4A). TFMG-induced abatement of in vitro permeability was significantly rescued by endothelial Tie2 knockdown and by blockage of the endothelial Tie2 receptor using anti-Tie2 antibody (Fig. 6a). This demonstrates a role for endothelial Tie2 in TFMG-induced vascular normalization.

In addition, immunofluorescence analysis revealed that TFMG treatment significantly enhanced ZO-1 levels, particularly at the cell-cell junctions of endothelial cells (Fig. 6b), confirming that the cellular tight junctions were increased when permeability was inhibited by the PCNs. To identify the Tie2 signaling pathways that increase cell junctional proteins, we performed a Western blot analysis. The results revealed that TFMG treatment of EC monocultures caused significant increases in Tie2 phosphorylation and its downstream target, Akt (Fig. 6c). This suggests that Akt activation results from Tie2 activation following TFMG-induced PAR-1/PAR-3 heterodimerization. We found that phosphorylated FoxO3a at S253 was increased by TFMG treatment, whereas FoxO3a levels were decreased, indicating that it is targeted for degradation by phosphorylation (Fig. 6c). We also observed a significant increase in the level of Rho kinase (ROCK-1), a downstream target of Rho-GTPase, and an increase in the level of the tight-junction protein zonula occludens-1 (ZO-1) following TFMG treatment of endothelial cells compared with untreated cells (Fig. 6c). To confirm the hypothesis that TFMG induces vascular normalization by increasing PAR-1/PAR-3 interaction and Tie2 activation in vivo, we performed an IHC analysis of tumor tissues. We found that phosphorylated Tie2 was upregulated by TFMG where endothelial PAR-3 was expressed (Fig. 6d). In addition, the interaction between PAR-1/PAR-3 in CD31-expressed endothelial cells was increased by TFMG treatment compared with the control in tumor tissues (Fig. 6e), suggesting that the PAR-1/PAR-3 interaction results in Tie2 activation and is involved in the vascular normalization by the PCNs.

As previously reported, PAR-1 interaction with PAR-3 alters the PAR-1/G_α13_, but not PAR-1/G_αq_, binding conformation, and thereby favors a distinct G_α13_-activated downstream pathway [32]. To further elucidate which G-protein isoform is involved in Tie2 activation by TFMG, we examined Tie2 activation by knocking down several G-proteins with siRNA in ECs. G_α13_, G_αi_, and G_αq_ siRNA exhibited more than 60% efficacy in suppressing gene expression at 48 h post-transfection (Fig. S4B). Activation of Tie2 and Akt was induced by TFMG and reduced in the absence of the G_α13_ G-protein (Fig. 6f), suggesting that Gα_13_ mediates Tie2 activation by TFMG. Taken together, these results indicate that PCN-induced vessel normalization depends on endothelial Tie2-induced tightening of endothelial junctions which results in reduced vascular permeability (Fig. 6g).

### Pericyte Tie2 has no direct effects on TFMG-induced vascular normalization

While pericyte-expressed Tie2 has been suggested to play a vital role in controlling tumor-associated angiogenesis and tumor growth [38], modest expression of PARs has also been associated with pericytes [39–41]. Therefore, we examined whether pericyte Tie2, in addition to endothelial Tie2, has any direct effect on TFMG-induced vascular normalization. To investigate the involvement of pericyte Tie2 in TFMG-induced reduction of vascular permeability, we established a PC and a pericyte-endothelial cancer cell (PEC) co-culture system (Fig. S5A and S5B). TFMG treatment did not inhibit in vitro permeability in the PC co-culture system (Fig. S5C), whereas TFMG treatment induced significant inhibition of in vitro permeability in the PEC co-culture system (Fig. S5D). In addition, siRNA-mediated silencing of pericyte PAR-1, PAR-2, PAR-3, or Tie2 resulted in no significant changes in TFMG-induced permeability reduction (Fig. S5D). These results indicate that there are no direct effects of pericyte PARs or Tie2 in TFMG-induced vascular normalization. In addition, TFMG treatment of pericyte monocultures did not result in the activation of pericyte Tie2, nor the activation of the downstream target Akt (Fig. S5E). This further supports the finding that TFMG-induced vascular normalization is not associated with pericyte Tie2.

### PCNs enhance the antitumor effects of chemotherapeutic agents in the LLC tumor model

**Fig. 7:**
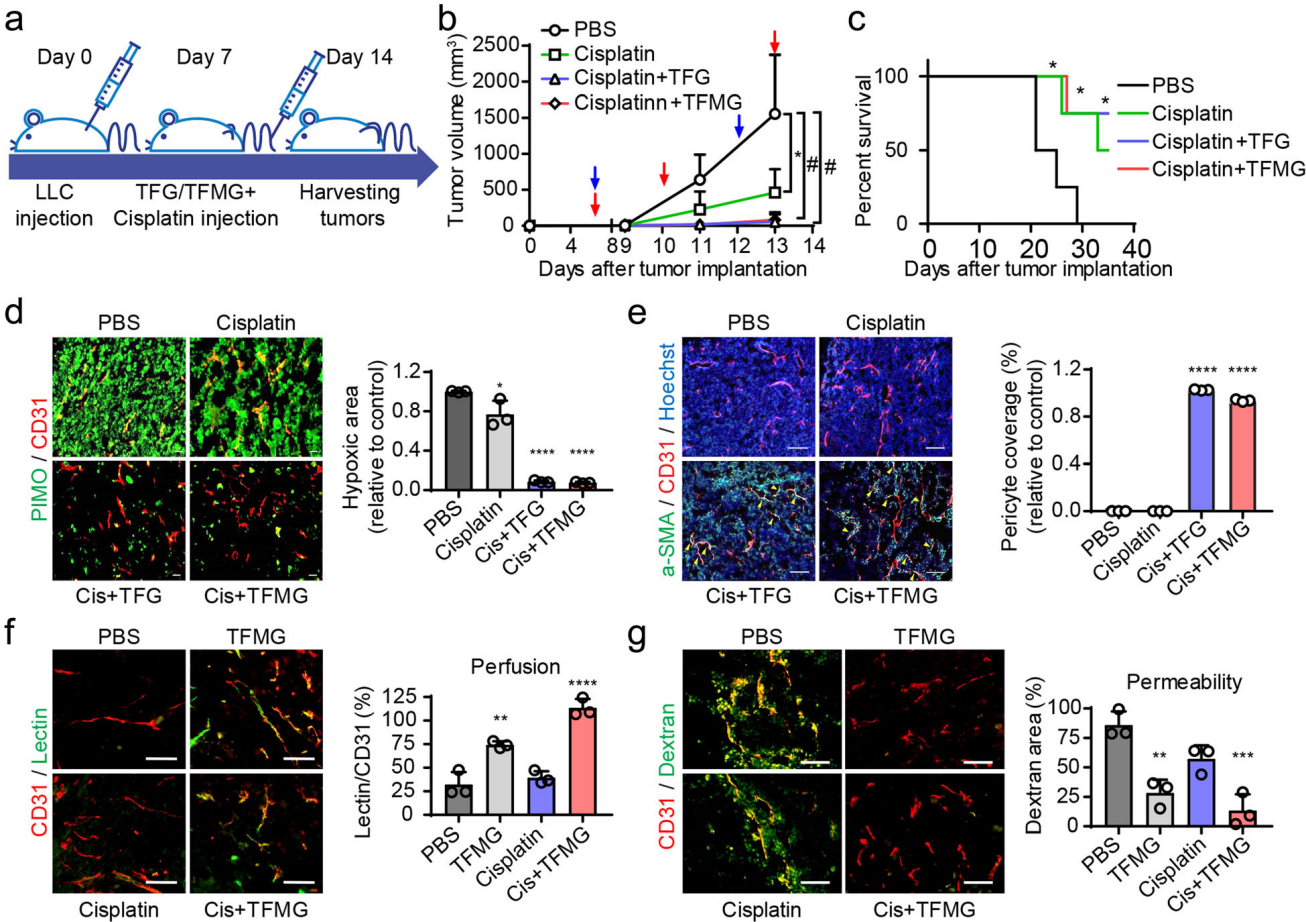
TFG and TFMG enhance the chemotherapeutic efficacy of cisplatin in LLC tumor-bearing mice. **a** Schematic diagram depicting the LLC allograft model development and treatment schedule. LLC tumor-bearing mice, following LLC cell inoculation, were administered intravenous injections of vehicle control (PBS) on days 7, 10, and 13, or cisplatin (3 mg/kg) on days 7 and 12, with or without TFG (10 nmol/kg) or TFMG (10 nmol/kg) on day 7s, 10, and 13. Tumors were harvested on day 14. **b** Tumor growth pattern in LLC tumor-bearing mice administered PBS, cisplatin, cisplatin and TFG, or cisplatin and TFMG. The red arrowheads represent the time points of TFG/TFMG administration, while the blue arrowheads represent the time points of cisplatin administration. Tumor volumes in each group at days 7, 9, 11, and 13 were calculated (*n* = 8). *P < 0.001; ^#^*P* < 0.0001 vs. PBS control. **c** Survival curves of LLC tumor-bearing mice. **d** Confocal images of tumor sections showing hypoxic regions in the center of the LLC tumors stained with Hypoxicprobe™ (green, PIMO, hypoxic areas) and CD31 (red, blood vessels) (*n* = 4). Scale bars: 100 μm. **e** Confocal images of tumor sections showing α-SMA+ pericyte coverage on tumor vessels. Frozen tumor sections were stained with α-SMA (green, pericyte), CD31 (red, blood vessels), and Hoechst 33258 (blue) (*n* = 4). Scale bars: 100 μm. **f** Mice bearing LLC tumor grafts were intravenously injected with (100 nmol/mouse) TFMG or PBS every other day three times. On the second day after the last injection, the tumor grafts were removed and sectioned (10 μm) while frozen. The endothelium was visualized using an antibody against CD31. After treatment, the mice were intravenously injected with 100 μg of Dylight488-lectin. Approximately 30 min later, tumor grafts were collected (*n* = 4). **g** To measure the permeability of tumor blood vessels after treatment, mice were intravenously injected with 2.5 mg FITC-dextran followed by 30 min of circulation. The tumor grafts were removed after heart perfusion (*n* = 3). The quantitations in (**d–g**) were conducted using ImageJ software. Data information: Data are presented as the mean ± SD. Significant enrichment: *P < 0.05; **P < 0.01; ***P < 0.001; ****P < 0.0001 (**c**–**g**) (two-way ANOVA in (**b**); Mantel-Cox test in (**c**); and Sidak’s multiple comparisons test)

Tumor vessel normalization is expected to not only reduce metastasis but also to improve delivery of chemotherapeutic drugs by enhancing perfusion [42, 43]. Based on the effects of the PCNs in reversing the functional impairment of the disorganized tumor vessels, we investigated whether PCNs enhanced the delivery and antitumor effects of chemotherapeutic agents. To examine the synergistic effects of PCNs with chemotherapeutic agents, TFMG was administered individually or co-administered with varying doses of cisplatin or doxorubicin to LLC tumor-bearing mice, and their respective antitumor effects were evaluated against vehicle-administered control mice. We found that a combined treatment of TFG or TFMG with cisplatin or doxorubicin enhanced the additive antitumor effects of either cisplatin (Figs. 7a, b; S6A; and S6B) or doxorubicin (Fig. S6C and S6D), leading to significant inhibition of tumor progression, not only compared with the control but also compared with cisplatin or doxorubicin monotherapy. Cisplatin combined with TFG or TFMG markedly enhanced the survival relative to the control or cisplatin monotherapy (Fig. 7c). In addition, cisplatin coadministration with TFG or TFMG significantly reduced the hypoxic area of tumors (Fig. 7d, PIMO), while significantly enhancing the pericyte coverage of the tumor blood vessels (Fig. 7e).

Next, to confirm the role of PCNs in normalizing the tumor vasculature, we assessed vascular perfusion using DyLight 488-conjugated lectin [44]. TFMG as well as cotreatment with cisplatin increased perfused lectin to the tissues (Fig. 7f). Additionally, the leakage of FITC-dextran from the vessels to the tissues [45] was significantly decreased by treatment with TFMG or the cisplatin combination (Fig. 7g). To compare histological alterations induced by cisplatin or antiangiogenic agents, we performed H&E staining of the tumor tissues following treatment with cisplatin or bevacizumab. We found a well-organized morphology in TFMG- or bevacizumab-treated tissue, but not in the cisplatin-treated group. Many dead cells were found in the tissues of the combined cisplatin plus TFMG group compared with the control group (Fig. S6E), suggesting that the cytotoxic activity of cisplatin was increased through restored perfusion by TFMG. These results suggest that PCNs increase the chemotherapeutic effects of cisplatin through normalization of the tumor vessels.

## Discussion

Tumors are not properly perfused due to destabilization of vascular structure and increased interstitial fluid pressure, which result in extreme hypoxia and impaired drug delivery. These alterations, in turn, enhance tumor progression, tissue invasion, metastasis, and resistance to chemotherapy [1]. Tumor vascular normalization, which is aimed at increasing perfusion and oxygenation, is associated with better clinical responses in subsets of diverse cancer patients [2]. In the present study, we demonstrated that ferritin-based protein C nanoparticles exhibited antitumor and anti-metastatic effects through tumor vascular normalization and antitumorigenic immune reprogramming in mouse models. The PCNs, TFG and TFMG, induced tumor vascular normalization by PAR-1/PAR-3 heterodimerization and Tie2 activation, which stabilized the vascular tight junctions through the Akt-FoxO3a signaling axis. The PCNs increased the number of cytotoxic CD8^+^ T lymphocytes and CD68^+^iNOS^+^ M1-like macrophages, induced minimal changes in CD4^+^ T lymphocytes, and decreased CD68^+^/Arg-1^+^ M2-like macrophages in tumor tissues. In addition, PCNs enhanced the chemotherapeutic effects of cisplatin by increasing blood perfusion and decreasing vascular permeability. These results suggest that the PCNs represent an efficient antiangiogenic therapeutic option to avoid the side effects of conventional angiogenic therapy or chemotherapy for treatment of advanced solid tumors.

The PCNs induced a significant inhibition of tumor growth both in an LLC mouse model and a spontaneous *MMTV*-*PyMT* breast tumor model, increased pericyte coverage, and reduced hypoxia in tumor sections. Vascular permeability drives (or vice versa) tumor-induced angiogenesis, inflammatory cell infiltration, and tumor extravasation, which ultimately increases metastatic potential [3, 4]. Hence, tumors strive to overcome the microenvironmental stress of vascular permeability in order to alleviate excessive angiogenesis and tumor extravasation [24]. Interestingly, increased blood flow into the tumor by normalization provides sufficient oxygen supply and increased drug delivery resulting in cancer cell death by immune cell penetration [10, 21]. Hypoxic areas in xenograft or metastatic tumors were reduced by TFG/TFMG treatment in mouse models, suggesting that vascular permeability is decreased while perfusion is increased by the PCNs.

Hypoxia causes immunosuppression by recruiting and activating immune suppressor cells such as regulatory T (Treg) cells and TAM cells [24]. The PCNs promoted an increased infiltration of cytotoxic T cells, but decreased regulatory T cells. The polarization of TAM and T cell infiltration by the PCNs may be induced by the disappearance of hypoxic regions in tumor tissues. Since an abnormal vasculature is advantageous for elevated hypoxia, defective immune cell trafficking, and tumor malignancy [10], our new therapeutic approach for converting the abnormal vasculature to a normal vasculature using PCNs is a powerful treatment option for advanced solid tumors.

EPCR binding activity is obtained by the Gla domain and PAR-1 cleavage activity is obtained by the TRAP domain of the PCNs. After cleavage of extracellular PAR-1 at the R46 residue [46], heterodimerization of PAR-1 with PAR-3 may occur, leading to Tie2 recruitment and activation. Here, we demonstrated that PCNs simultaneous occupy EPCR and activates the Tie2 receptor resulting in endothelial tight junctions. TFMG-induced inhibition of in vitro permeability in EC co-cultures was effectively antagonized by siRNA-mediated endothelial Tie2 silencing as well as antibody-mediated endothelial Tie2 blockage, which is implicated in Tie2 activation. This further led to increased levels of tight-junction marker proteins in the endothelial cells. As Tie2 activation has been shown to be an important event in the normalization of tumor vessels [21] as well as in sepsis [47], targeting EPCR and Tie2 by PCNs represents a highly efficient and effective way to treat cancers.

To further identify the signaling pathway underlying PCN antitumor activity, we investigated the EPCR/Tie2-mediated signaling pathway in endothelial cells. G_α13_ G-protein is involved in the activation of RhoGTPase nucleotide exchange factors (RhoGEFs), the downstream target of PAR-1/PAR-3, which leads to activation of the small monomeric GTPase, RhoA, and other downstream effectors [48]. In addition, Akt promotes endothelial barrier protection through a FoxO-mediated pathway, regulating turnover of tight-junction proteins, such as ZO-1 [49]. The PCN-activated G_α13_/RhoA may phosphorylate Tie2 and induce the activation of Akt and FoxO3a, resulting in an enhancement of tight junctions by increasing ZO-1 (Fig. 6e). These results suggest that the Tie2/Akt/FoxO3a signaling axis mediated through PAR-1/PAR-3 heterodimerization is an essential signaling pathway for the normalization of tumor vessels by the PCNs.

Excellent clinical results have been commonly associated with nanoparticle drug delivery systems exhibiting high clinical efficacy and significantly reduced adverse effects [50, 51]. Ferritin nanoparticles are useful platforms for therapeutic agents. They can be chemically modified to impart functionalities within their interior cavities or on their surfaces because of their unique architecture [17]. Unlike most other proteins, ferritin possesses unique properties, such as high solubility, stability, abundance in blood, and low toxicity, which motivates studies on its use as an ideal nanoplatform with many applications including disease therapy and drug delivery [18]. Here, we used ferritin as a template to encapsulate and deliver PCNs as a potential cancer therapeutic. In addition, we showed that concomitant administration of cisplatin with PCNs significantly enhanced antitumor activity and survival because of the capability of PCNs to normalize abnormal tumor vessels. If anticancer drugs can be used at lower doses while maintaining efficient delivery, reduced side effects would be expected [52]. Therefore, combination therapy using anticancer drugs with the PCNs may be an excellent therapeutic strategy.

In the present study, we showed that PCNs could be used to enhance normalization of the tumor vasculature and increase the immune response in the tumor microenvironment. The underlying mechanisms involved in PCN-induced vascular normalization involve endothelial PAR-3 and Tie2 activity following EPCR binding and PAR-1 activation. PAR-1 activation by the PCNs led to PAR-1/PAR-3 heterodimerization, which subsequently induced activation of the G_α13_-RhoA GTPase and Tie2 signaling. This ultimately resulted in increased recruitment of tight-junction proteins through FoxO3-mediated transcription at endothelial cell junctions. In addition, PCN-induced Tie-2 activation promotes pericyte coverage of the tumor vasculature and hypoxic regions even at core regions of the tumor.

## Conclusions

Our findings reveal that tumor vascular normalization by PCNs may provide a breakthrough in the modern approach to cancer therapy. Further studies with respect to the pharmacological action of the PCNs may provide a new strategy for the inhibition of tumor angiogenesis and effective cancer therapy.

## Supplementary information

**Additional file 1: Figure S1**. Characterization of TFG and TFMG *in vitro*. **Figure S2**. Characterization of TFG and TFMG *in vivo*. **Figure S3**. Quantitative RT-PCR analysis of ECs transfected with indicated siRNAs. **Figure S4**. Quantitative RT-PCR analysis of ECs transfected with indicated siRNAs. **Figure S5.** Pericyte Tie2 has no direct role in TFMG-induced vascular normalization. **Figure S6**. Histological analysis of tumor tissues in LLC tumor allograft mice. **Table S1**. Pharmacokinetic parameters of TRAP and TFMG. **Table S2**. Oligonucleotides used for qRT-PCR.

## Data Availability

All data generated or analyzed during this study are included in this article and its additional files.

## Abbreviations

DTT: Dithiothreitol
EC: Endothelial cell/cancer cell
EPCR: Endothelial protein C receptor
FA: Formaldehyde
Gla: γ-Carboxyglutamic acid
IHC: Immunohistochemical
IPTG: Isopropyl-β-d-1-thiogalactopyranoside
LLC: Lewis lung carcinoma
PAR-1: Protease-activated receptor-1
PBS: Phosphate-buffered saline
PC: Pericyte/cancer cell
PCNs: Protein C nanoparticles
PEC: Pericyte/endothelial cell/cancer cell
PFA: Paraformaldehyde
qRT-PCR: Quantitative real-time polymerase chain reaction
RhoGEFs: RhoGTPase nucleotide exchange factors
SMA: α-Smooth muscle actin
TAM: Tumor-associated macrophages
TRAP: Thrombin receptor agonist peptide
Treg: Regulatory T
VEGF: Vascular endothelial growth factor
ZO-1: Zonula occludens-1
