# Supplementary material for: Tie2-mediated vascular remodeling by ferritin-based protein C nanoparticles confers antitumor and anti-metastatic activities
Source: J Hematol Oncol. 2020 Sep 14;13:123. doi: 10.1186/s13045-020-00952-9 (PMC7489044; doi:10.1186/s13045-020-00952-9)

**Supplemental Information**

**Tie2-mediated vascular remodeling by ferritin-based protein C nanoparticles confers antitumor and anti-metastatic activities**

Young Sun Choi^1,3,¶^ Hyeonha Jang^1,3^, Biki Gupta^2,3,‖^, Ji-Hak Jeong ^2,3^, Yun Ge^1,2^, Chul Soon Yong^4^, Jong Oh Kim^4^, Jong Sup Bae^1,2^, Im-Sook Song^1,2,3^, In-San Kim^5^, and You Mie Lee^1,2,3, *^

^1^BK21 Plus KNU Multi-Omics Creative Drug Research Team, ^2^Research Institute of Pharmaceutical Sciences, ^3^Vessel-Organ Interaction Research Center, VOICE (MRC), Department of Molecular Pathophysiology, College of Pharmacy, Kyungpook National University, Daegu, 41566, Republic of Korea. ^4^College of Pharmacy, Yeungnam University, Gyeongsan 38541, Republic of Korea. ^5^Biomedical Research Institute, Korea Institute of Science and Technology, Seoul, 02792, Republic of Korea

*** Correspondence to:**

You Mie Lee, Ph.D. Professor,

Vessel-Organ Interaction Research Center, VOICE (MRC), Department of Molecular Pathophysiology, College of Pharmacy, Kyungpook National University, Daegu, 41566, Republic of Korea.

Tel: +82-53-950-8566, Fax: +82-53-950-8557, E-mail: [lym@knu.ac.kr](mailto:lym@knu.ac.kr)

ORCID: <https://orcid.org/0000-0002-5756-7169>

**
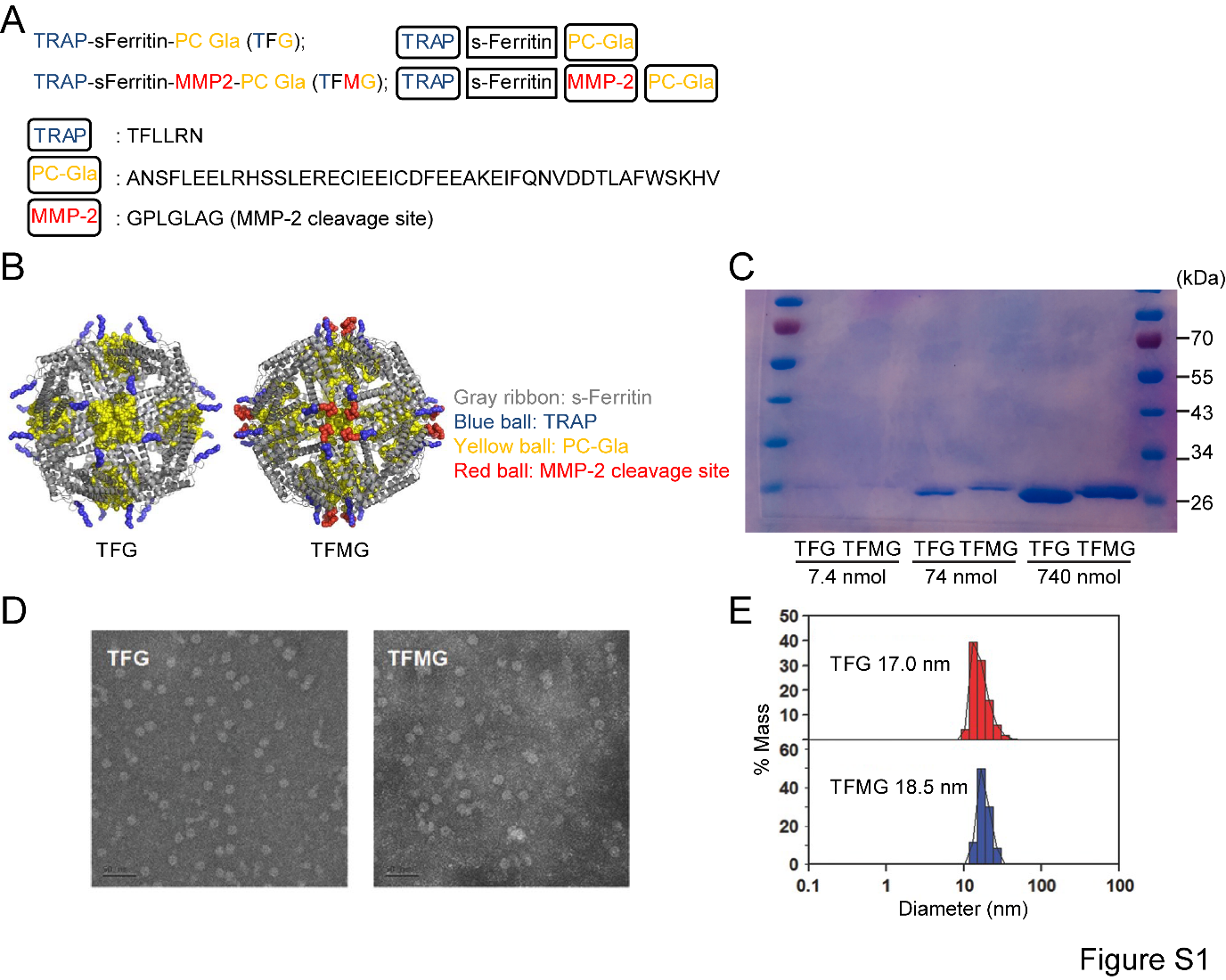
**

**Figure S1. Characterization of TFG and TFMG *in vitro*.**

**(A)** Schematic diagram of TFG and TFMG. TRAP peptide and PC-Gla with or without an MMP-2 cleavage site were inserted into short ferritin (sFn).

**(B)** 3D models of TFG and TFMG were prepared by PyMol^*^. The structures of TRAP and PC-Gla with or without an MMP-2 cleavage site were calculated based on the crystal structure of sFn. ^*^Schrödinger LLC (The PyMOL Molecular Graphics System, Version 1.3r1, 2010, the JyMOL Molecular Graphics Development Component, Version 1.0, 2010, and the AxPyMOL Molecular Graphics Plugin for Microsoft PowerPoint, Version 1.0, 2010).

**(C)** Photographic image of SDS-PAGE gel electrophoresis analysis of TFG and TFMG.

**(D)** Transmission electron microscopes (TEM) images. Scale bars represent 50 nm. (*Adv Mater.* 2015;27(42):6637-43)

**(E)** Size distribution measurements of TFG and TFMG assemblies. (*Adv Mater.* 2015;27(42):6637-43)


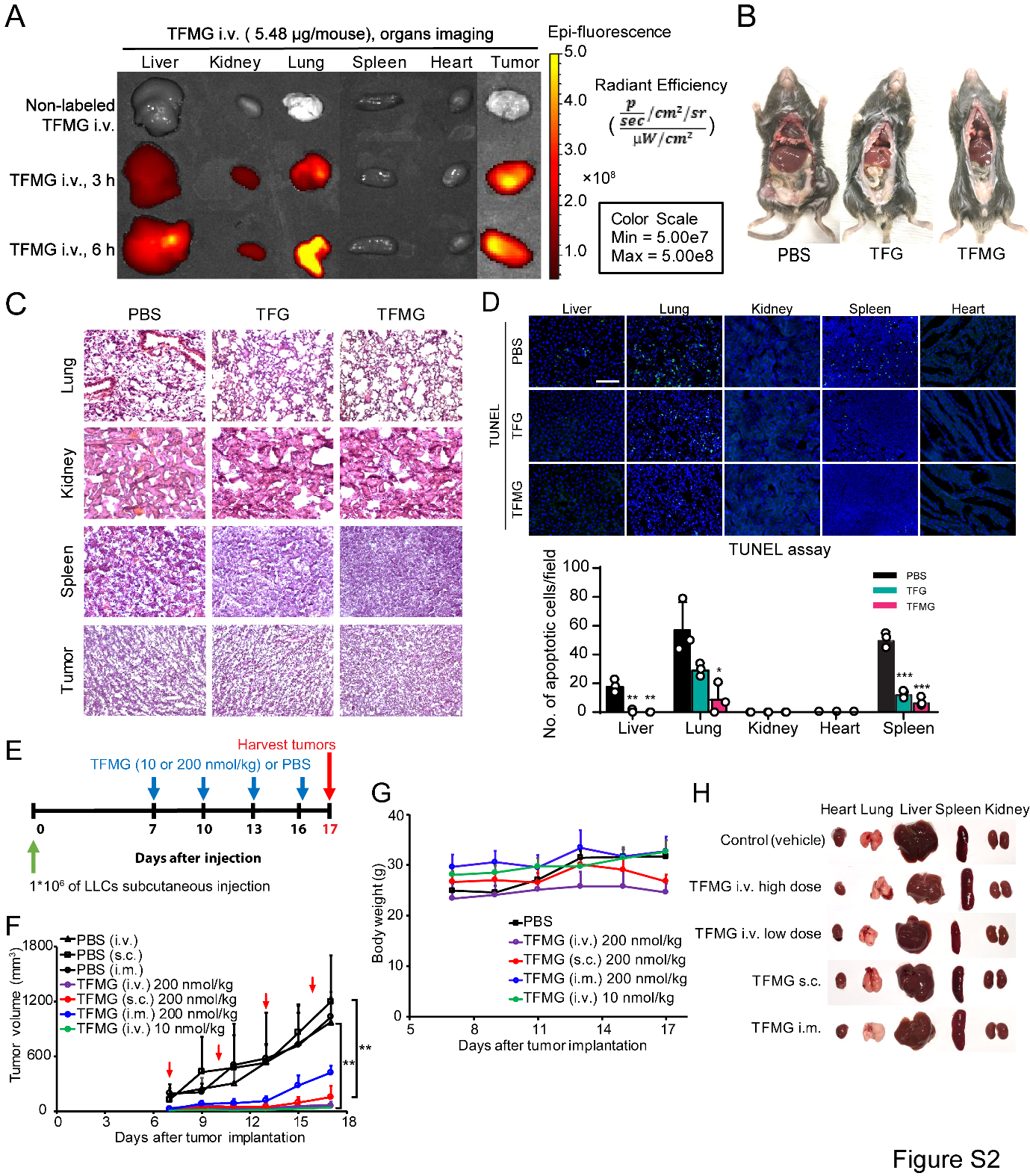


**Figure S2. Characterization of TFG and TFMG *in vivo*.**

**(A)** Analysis for in vivo delivery of TFMG. After injected intravenously with FNI-675 NHS ester-conjugated TFMG (5.48 μg/mouse), 3 and 6 h after injection fluoresce images of various organs including tumors were analyzed by IVIS from LLC allograft tumor bearing mice.

**(B)** Gross morphology of internal organs after cut the abdomen open in PBS-treated control and TFG- or TFMG- treated mice with the same treatment schedule to the experiment in Figure 1.

**(C)** Histological analysis for different organs of TFMG-treated mice. Various organs from the same mice as (B) were stained with H&E for examination of gross organ toxicity. Magnification (100$\times$, 200$\times$).

**(D**) Apoptotic cells were detected by the TUNEL in liver, lung, kidney, spleen and heart tissue sections. DNA fragmentation in apoptotic cells are in green, and nuclei of cells are in blue (DAPI). Scale bar: 50 µm. Data are presented as mean ± SD (n = 3); ^*^*P* < 0.05, ^**^*P* < 0.01, and ^***^*P* < 0.001 vs PBS control.

**(E)** Schematic diagram depicting development of LLC allograft models and treatment schedule of TFMG. LLC tumor-bearing mice, following LLC cells inoculation, were administered with TFMG or PBS (vehicle) at indicated concentrations by intravenous (i.v.), subcutaneous (s.c), or intramuscular (i.m.) injections on day 7, 10, and 13, and the tumors were sampled on day 17. Statistical analysis was done using two-way ANOVA. ***P <* 0.01.

**(F)** Pattern of tumor growth in LLC tumor-bearing mice from (D). The red arrowheads represent the point of TFMG administration. Data are presented as mean ± SD (n ≥ 9); ^**^*P* < 0.01 vs PBS control.

**(G)** Body weight patterns of LLC tumor-bearing mice from (D).

**(H)** Gross morphology of various organs of LLC tumor-bearing mice from (D).

**
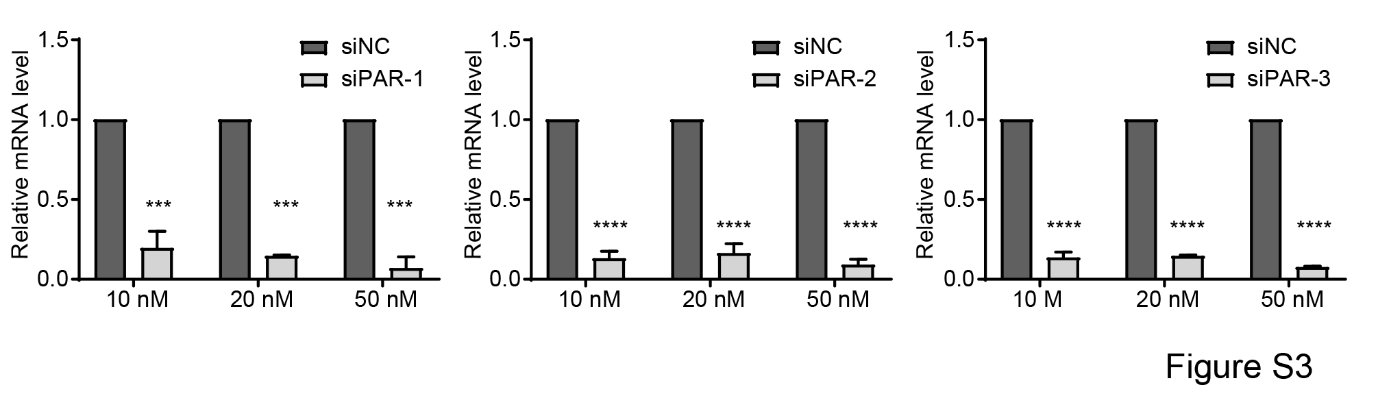
**

**Figure S3. Quantitative RT-PCR analysis of ECs transfected with indicated siRNAs.**

ECs were transfected with siPAR-1, siPAR-2, or siPAR-3 at 10, 20 and 50 nM for 48 h, respectively. Data are presented as mean ± SD (n = 3). ^***^*P* < 0.001; ^****^*P* < 0.0001 vs control siRNA.

**
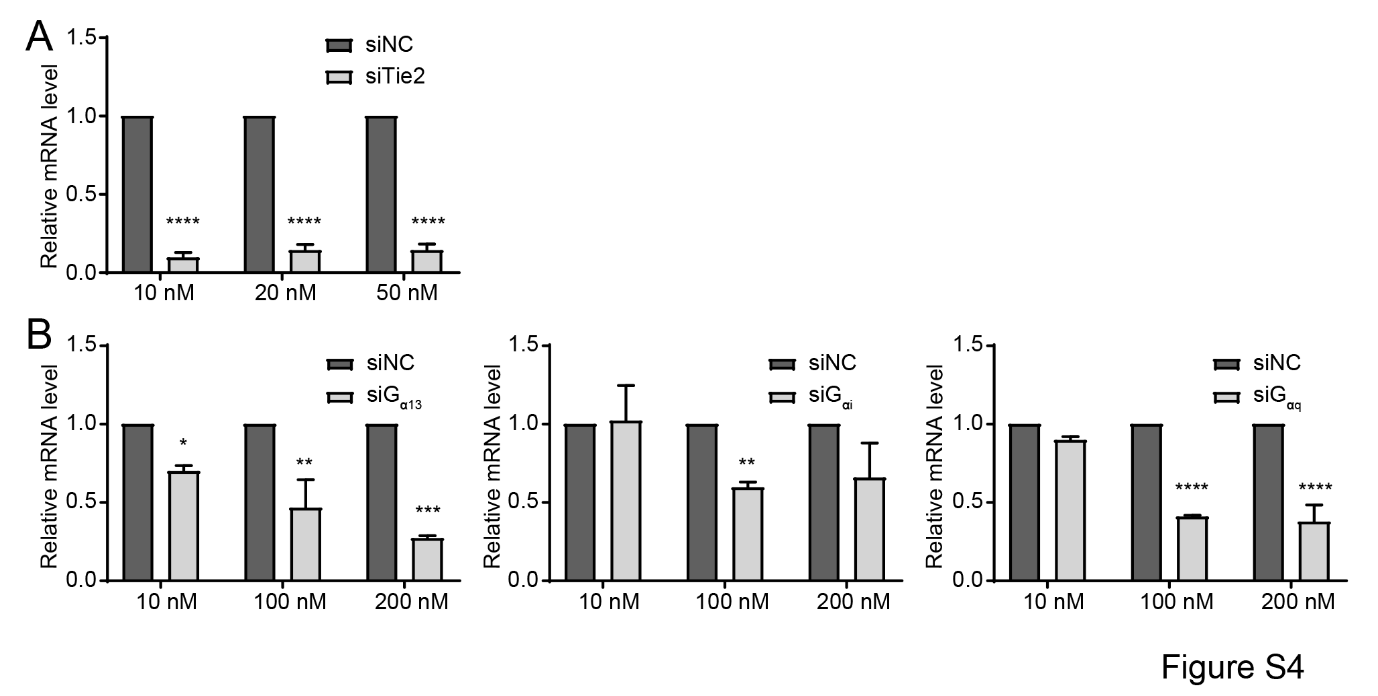
**

**Figure S4. Quantitative RT-PCR analysis of ECs transfected with indicated siRNAs.**

**(A)** ECs were transfected with siTie2 at 10, 20 and 50 nM concentration for 48 h, respectively.

**(B)** ECs were transfected with siGα13, siGαi, or siGαq at 10, 100 and 200 nM for 48 h, respectively. Data are presented as mean ± SD (n = 3). ^*^*P* < 0.05; ^**^*P* < 0.01; ^***^*P* < 0.001; ^****^*P* < 0.0001 vs control siRNA.

**
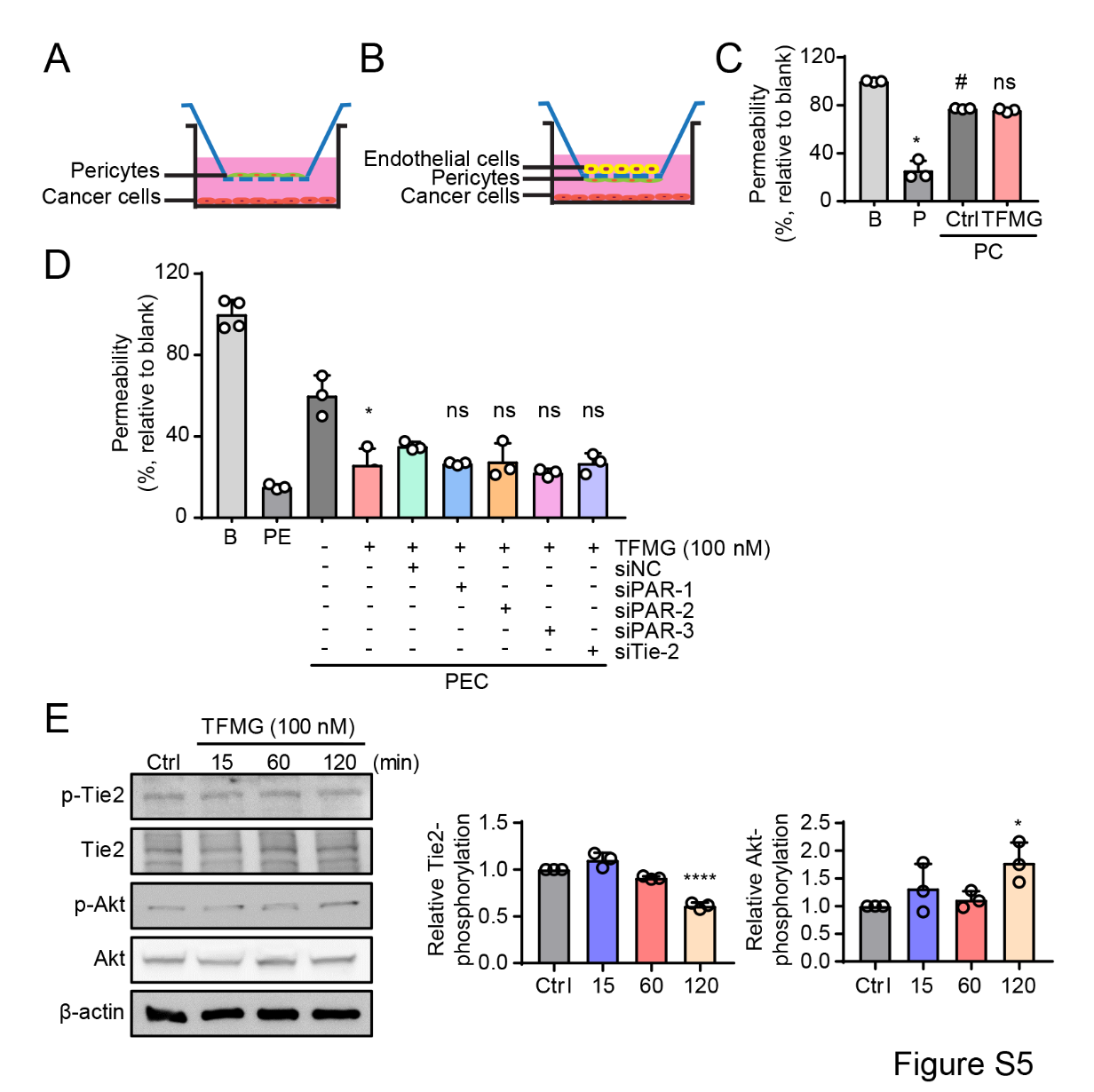
**

**Figure S5. Pericyte Tie2 has no direct role in TFMG-induced vascular normalization.**

**(A)** Schematic diagram of pericyte/cancer cell (PC) co-cultures. C3H/10T1/2, clone 8 (2.5 × 10^5^) cells were seeded over 12-well transwell membrane inserts while LLC (2.5 × 10^5^) cells were seeded inside the 12-well plates.

**(B)** Schematic diagram of pericyte/endothelial/cancer cell (PEC) co-cultures. C3H10T1/2, clone 8 (2.5 × 10^5^) cells were seeded on the lower surface of 12-well transwell membrane inserts while EA.hy926 (5 × 10^5^) cells were seeded on the upper surface of the, and LLC cells were seeded inside the 12-well plates.

**(C)** Permeability assay of TFMG using PC co-cultures. PC co-cultures were treated with TFMG (100 nM) and the permeability (%) of FITC-dextran was determined against untreated control co-cultures. B, blank (no C3H/10T1/2, no LLC); P, pericyte only (C3H/10T1/2 cells seeded onto the transwell membrane, no LLC); ^*^*P* < 0.0001 vs B; ^#^*P* < 0.0001 vs P; ns, no significant difference vs Ctrl (n = 3).

**(D)** Effects of siRNA-mediated pericyte PAR-1, PAR-2, PAR-3, and Tie2 silencing on TFMG-induced reduction of in vitro permeability. PEC co-cultures, with or without siRNA-mediated silencing of endothelial PAR-1, PAR-2 or PAR-3, were treated with 100 nM TFMG for 24 h, and the permeability (%) of FITC-dextran was determined against untreated control co-cultures. B, blank; PE, pericyte/endothelial cell co-culture; ^*^*P* < 0.001 vs untreated control PEC co-cultures; ns, no significant difference vs TFMG-treated PEC co-cultures with or without siNC silencing (n = 3).

**(E)** Western blot analysis to evaluate levels of p-Tie2, Tie2, p-Akt, and Akt in pericytes following TFMG treatment. C3H/10T1/2, clone 8 cell monocultures were treated with 100 nM of TFMG for 15, 60, and 120 min and levels of p-Tie2, Tie2, p-Akt, and Akt were determined by Western blotting; ^*^*P* < 0.05, and ^****^*P* < 0.0001 vs control.

**
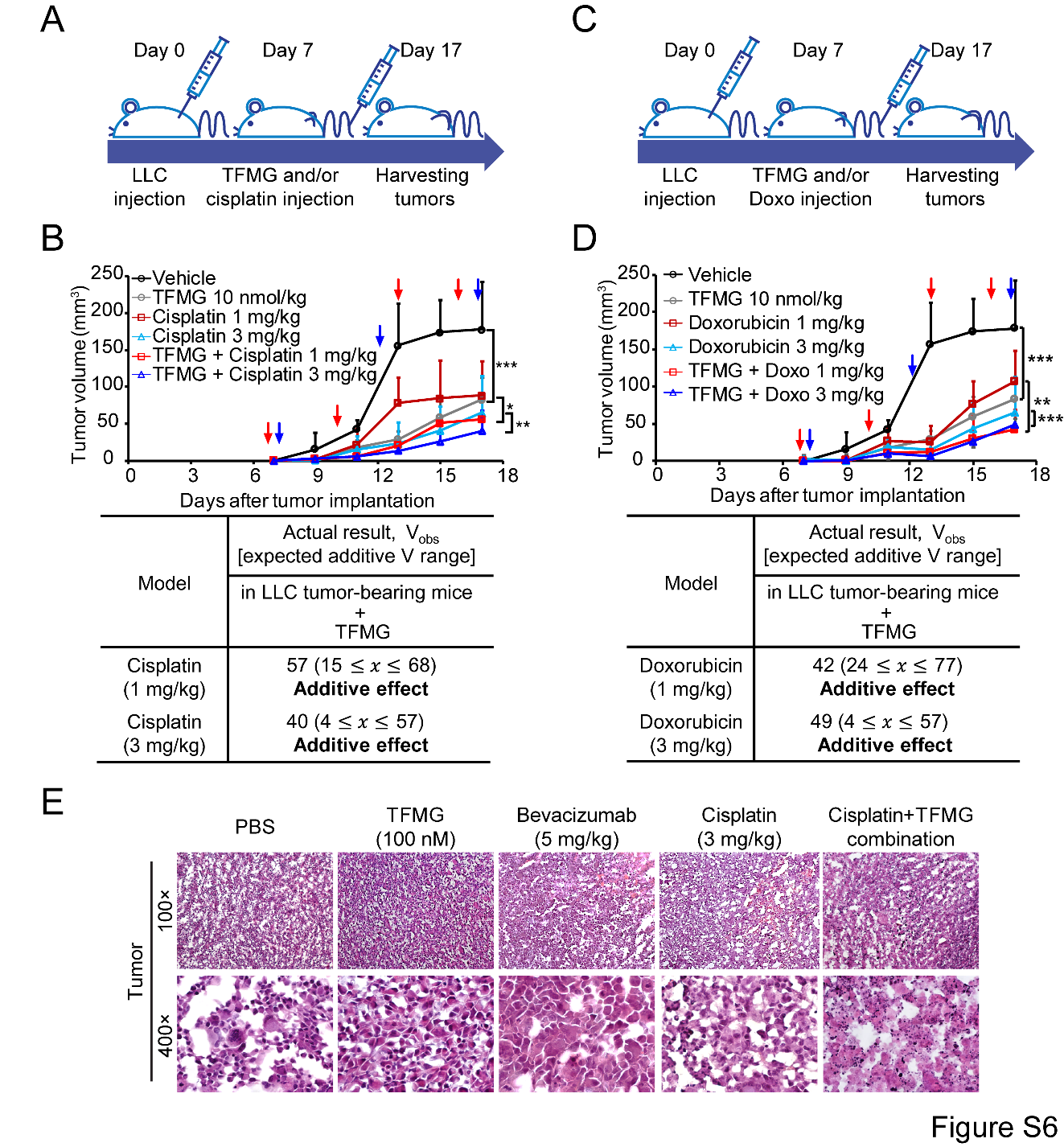
**

**Figure S6. Histological analysis of tumor tissues in LLC tumor allograft mice.**

**(A)** Schematic diagram depicting development of LLC allograft models and treatment schedule. LLC tumor-bearing mice, following LLC cells inoculation, were administered TFG (10 nmol/kg) and/or cisplatin (1 mg/kg, 3 mg/kg), or vehicle control (PBS) intravenously, and the tumors were sampled on day 17.

**(B****)** Pattern of tumor growth in LLC tumor-bearing mice from (A). Tumor volumes were measured every second day from day 7. The red arrowheads represent the point of TFMG administration, and the blue arrowheads represent the point of cisplatin administration (n ≥ 9). Observed mean tumor volume and expected additive tumor volume range, calculated via the Bliss independence model.

**(C)** Schematic diagram depicting development of LLC allograft models and treatment schedule. LLC tumor-bearing mice, following LLC cells inoculation, were administered TFMG (10 nmol/kg) and/or doxorubicin (1 mg/kg, 3 mg/kg), or vehicle control (PBS) intravenously, and the tumors were sampled on day 17.

**(D)** Pattern of tumor growth in LLC tumor-bearing mice from (C). Tumor volumes were measured every second day from day 7. The red arrowheads represent the point of TFMG administration, and the blue arrowheads represent the point of doxorubicin administration (n ≥ 9). Observed mean tumor volume and expected additive tumor volume range, calculated via the Bliss independence model.

**(E)** After treatment of TFMG, cisplatin, or bevacizumab, or cotreatment of TFMG and cisplatin with the same treatment schedule in Figure 7A, sectioned tumor tissues (5 µm) were analysed for H&E staining.

Data information: Data are presented as the mean $\pm$ SD. Significant enrichment: ^*^*P* < 0.05; ^**^*P* < 0.01; ^***^*P* < 0.001 (two-way ANOVA).

**Table S1. Pharmacokinetic parameters of TRAP and TFMG**


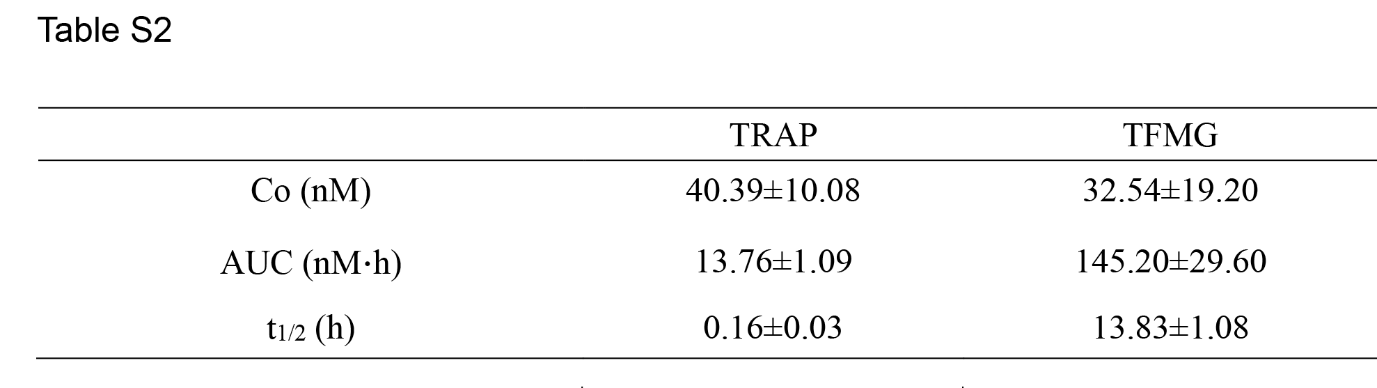


Parameters were calculated from the data in Figure 1B and 1C. Data represent the mean ± standard deviations from 3 three mice per group. Co: initial concentration; AUC: area under plasma concentration curve; t1/2: half-life.

**Table S2. Oligonucleotides used for qRT-PCR**


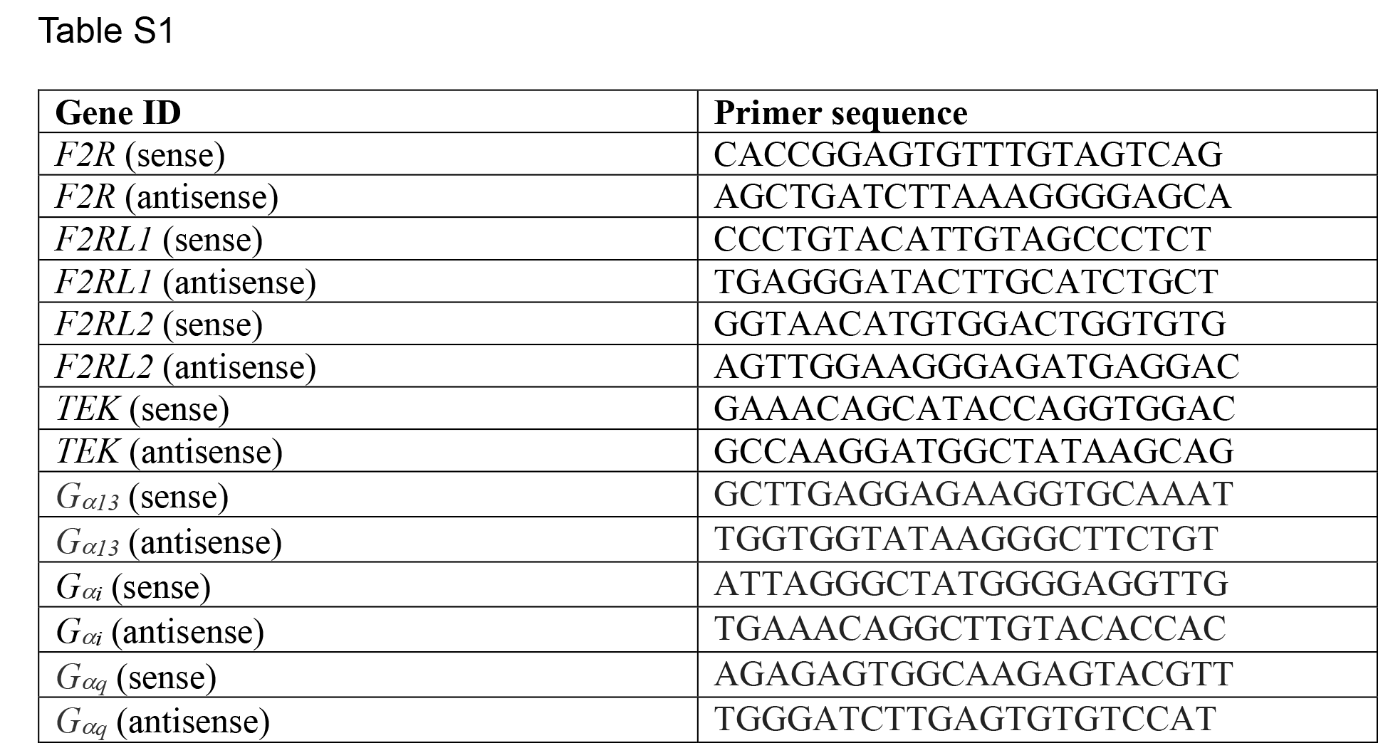

Supplement: Supplementary file 1 — Additional file 1: Figure S1. Characterization of TFG and TFMG in vitro. Figure S2. Characterization of TFG and TFMG in vivo. Figure S3. Quantitative RT-PCR analysis of ECs transfected with indicated siRNAs. Figure S4. Quantitative RT-PCR analysis of ECs transfected with indicated siRNAs. Figure S5. Pericyte Tie2 has no direct role in TFMG-induced vascular normalization. Figure S6. Histological analysis of tumor tissues in LLC tumor allograft mice. Table S1. Pharmacokinetic parameters of TRAP and TFMG. Table S2. Oligonucleotides used for qRT-PCR. [file 13045_2020_952_MOESM1_ESM.docx]
